# Portrait of *Candida albicans* Adherence Regulators

**DOI:** 10.1371/journal.ppat.1002525

**Published:** 2012-02-16

**Authors:** Jonathan S. Finkel, Wenjie Xu, David Huang, Elizabeth M. Hill, Jigar V. Desai, Carol A. Woolford, Jeniel E. Nett, Heather Taff, Carmelle T. Norice, David R. Andes, Frederick Lanni, Aaron P. Mitchell

**Affiliations:** 1 Department of Biological Sciences, Carnegie Mellon University, Pittsburgh, Pennsylvania, United States of America; 2 Department of Medicine, Section of Infectious Diseases, University of Wisconsin, Madison, Wisconsin, United States of America; 3 Department of Microbiology, Columbia University, New York, New York, United States of America; University of Toronto, Canada

## Abstract

Cell-substrate adherence is a fundamental property of microorganisms that enables them to exist in biofilms. Our study focuses on adherence of the fungal pathogen *Candida albicans* to one substrate, silicone, that is relevant to device-associated infection. We conducted a mutant screen with a quantitative flow-cell assay to identify thirty transcription factors that are required for adherence. We then combined nanoString gene expression profiling with functional analysis to elucidate relationships among these transcription factors, with two major goals: to extend our understanding of transcription factors previously known to govern adherence or biofilm formation, and to gain insight into the many transcription factors we identified that were relatively uncharacterized, particularly in the context of adherence or cell surface biogenesis. With regard to the first goal, we have discovered a role for biofilm regulator Bcr1 in adherence, and found that biofilm regulator Ace2 is a major functional target of chromatin remodeling factor Snf5. In addition, Bcr1 and Ace2 share several target genes, pointing to a new connection between them. With regard to the second goal, our findings reveal existence of a large regulatory network that connects eleven adherence regulators, the zinc-response regulator Zap1, and approximately one quarter of the predicted cell surface protein genes in this organism. This limited yet sensitive glimpse of mutant gene expression changes had thus defined one of the broadest cell surface regulatory networks in *C. albicans*.

## Author Summary

Most microorganisms adhere to surfaces in nature, leading to formation of complex communities called biofilms. Pathogen adherence to medical devices is the basis for device-associated infection. We have focused on the control of adherence in the fungal pathogen *Candida albicans.* We find that this process is under control of thirty transcriptional regulators. Our analysis of gene expression in regulatory mutants with altered adherence provides new understanding of the relationships among known regulators. In addition, we find evidence for a large regulatory network that connects one quarter of all cell surface protein genes.

## Introduction

Microorganisms naturally exist primarily in association with surfaces in communities called biofilms. Central to the formation of biofilms is the ability of microbial cells to adhere to substrates. Adherence mechanisms are diverse, and involve specific cell surface proteins (adhesins), more complex surface structures such as pili, and secreted extracellular matrix material [1]–[4]. Adherence is often found to be highly regulated, reflecting the need for biofilms to release cells in order to colonize new sites.

Biofilms are clinically significant as the basis for infections associated with implanted medical devices [5], [6]. Adherence of a pathogen to a device surface is a critical early step in formation of these biofilms. For device-associated biofilms, definition of the mechanisms that regulate cell-substrate adherence provides insight into how these biofilms form. That understanding may in turn suggest simple therapeutic or preventive strategies.

Our focus is the fungal pathogen *Candida albicans*, a natural commensal of our gastrointestinal and genitourinary tracts that is usually benign. It causes infections associated with venous catheters, urinary catheters, and several other implanted devices [7], [8]. Our overall understanding of *C. albicans* biofilm formation has expanded dramatically in recent years, and several regulators and effectors that contribute to biofilm formation are known [1], [9], [10]. Several key effectors have been identified among targets of transcription factors that are required for normal biofilm formation. The approach of using a transcription factor mutant to identify functional targets has proven particularly useful because many effectors are specified by duplicated genes or gene families [1].

In this study we focus on an early step in abiotic surface biofilm formation, the adherence of yeast form cells to a substrate. We find that this process is governed by over 10% of the *C. albicans* transcription factors, thus indicating that adherence is coupled to numerous regulatory signals. We use nanoString profiling [11] to analyze gene expression changes for all of these transcription factor mutants. Although nanoString probes cover only a portion of the transcriptome, the sensitivity exceeds that of microarrays [11]. In addition, the probes recognize RNA directly, avoiding possible bias from cDNA conversion [11]. Our findings reveal new connections between these regulators that we validate with functional assays. In addition, our results define a group of 37 cell surface protein genes that are coordinately regulated by twelve transcription factors. This newly discovered regulon may couple cell-substrate adherence to environmental signals.

## Results

### Regulators of substrate adherence

**Figure 1:**
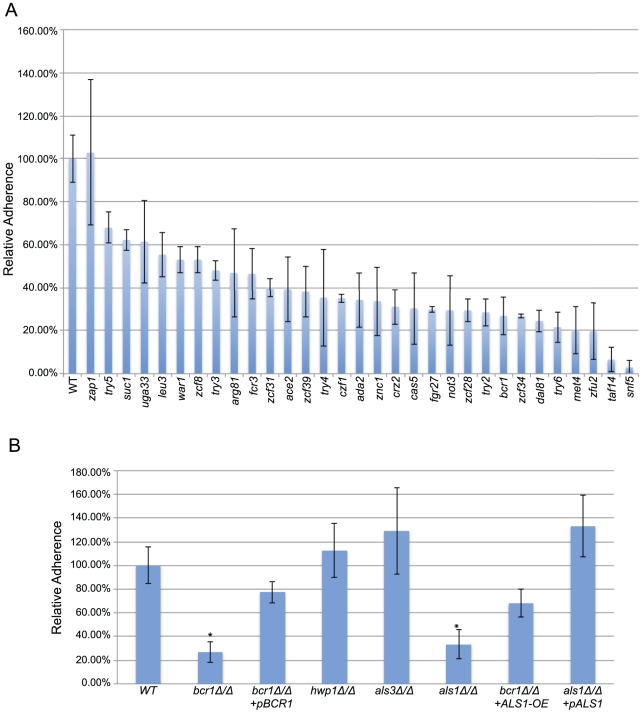
Adherence of wild-type and mutant strains. Adherence to silicone was measured in a Fluxion flow cell as described in Methods, and is expressed relative to the wild-type reference strain. Panel A. Transcription factor insertion mutants. The mutants presented had statistically significant decreases (p value ≤0.05) in adherence when compared to reference strain DAY286. The *zap1Δ/Δ* mutant is included for reference. Measurements indicate mean and standard deviation for 1–3 isolates, as indicated in Table S1 worksheet 1A. Panel B. Analysis of Bcr1 and its target genes. The wild-type strain DAY185 was used as a standard for comparison to mutants *bcr1Δ/Δ* (CJN702), *bcr1Δ/Δ+pBCR1* (CJN698), *hwp1Δ/Δ* (CAH7-1A1E2), *als3Δ/Δ* (CAYF178U), *als1Δ/Δ* (CAYC2YF1U), *bcr1Δ/Δ+ALS1-OE* (CJN1144), and *als1Δ/Δ+pALS1* (CAYC1). Asterisks indicate statistically significant decreases in adherence compared to the wild-type strain.

We assayed 197 transcription factor insertion mutants for altered cell-substrate adherence in a quantitative flow-cell assay, using a silicone (poly-dimethyl siloxane) substrate. We identified mutants in 30 genes with significantly reduced adherence compared to the wild type strain (Figure 1A; Table S1). We used three approaches to confirm that the known insertion mutation in each strain, rather than spurious mutations, caused its adherence defect (summarized in Table 1 under “Confirmation approaches”). First, for 26 genes, independent insertion mutant isolates were available. We assayed adherence of those strains, and found that they also displayed reduced adherence (Table S1). Second, for 25 genes, independently constructed deletion mutants were obtained in the BWP17 or SN152 strain backgrounds [12]. Adherence assays of those strains also confirmed the mutants' reduced adherence (Supplemental Tables S1B, S1C). Third, for 19 genes, we complemented the mutation by introducing a wild-type copy of the affected gene into the respective insertion or deletion mutant; we observed that wild-type levels of adherence were restored (Table S1). In total, our results verify the adherence defects for 29 of the mutants (Table 1).

**Table 1:** Summary of adherence mutant properties.

			Confirmation approaches						
orf19a	Candida Gene^a^	S.c. ortholog/Best Hit/TF Class^b^	Independent insertion mutant Isolates	SN152 Deletion^c^	BWP17 Deletion^d^	Comple-mentation	Mutant Relative Adherence^e^	*Mutant+ZAP1-OE* Relative Adherence^e^	Significant Gene Expression Changes^f^
19.6124	*ACE2*	*ACE2*	Y	Y	Y	Y	39%	filamentous	89
19.2331	*ADA2*	*ADA2*	Y	N	Y	Y	20%	35%	138
19.4766	*ARG81*	*ARG81*	Y	Y	Y	Y	23%	110%	91
19.723	*BCR1*	*YPL230W*	Y	Y	Y	Y	27%	23%	155
19.4670	*CAS5*	*MIG2*	Y	Y	N	N	24%	32%	88
19.2356	*CRZ2*	*CRZ1*	Y	Y	Y	Y	14%	37%	54
19.3127	*CZF1*	*UME6*	Y	Y	N	N	21%	51%	68
19.3252	*DAL81*	*DAL81*	Y	Y	N	Y	37%	90%	50
19.3193	*FCR3*	*YAP3*	Y	Y	N	Y	19%	100%	59
19.6680	*FGR27*	*ASG1*	Y	Y	N	N	40%	74%	133
19.4225	*LEU3*	*LEU3*	Y	Y	N	N	18%	35%	67
19.5312	*MET4*	*MET4*	Y	N	N	N	26%	22%	98
19.2012	*NOT3*	*NOT3*	Y	N	N	N	32%	23%	57
19.5871	*SNF5*	*SNF5*	Y	N	Y	Y	3%	32%	178
19.7319	*SUC1*	*MAL13*	Y	Y	N	Y	48%	93%	53
19.798	*TAF14*	*TAF14*	N	N	N	N	15%	20%	80
19.4062	*TRY2*	CCCH ZF	Y	N	N	Y	28%	99%	80
19.1971	*TRY3*	C3HC4 ZF	N	N	N	Y	51%	83%	76
19.5975	*TRY4*	*ADR1*	Y	Y	N	Y	41%	35%	93
19.3434	*TRY5*	*YGR067C*	Y	Y	N	Y	42%	48%	100
19.6824	*TRY6*	HLH motif	Y	Y	N	Y	27%	24%	51
19.7317	*UGA33*	*UGA3*	Y	Y	N	Y	25%	95%	122
19.1035	*WAR1*	*WAR1*	Y	Y	N	N	24%	20%	16
19.4767	*ZCF28*	*ECM22*	Y	Y	Y	Y	23%	111%	64
19.5924	*ZCF31*	ZN(2)-C6	N	Y	N	N	25%	11%	40
19.6182	*ZCF34*	*PDR1*	Y	Y	N	Y	29%	48%	108
19.7583	*ZCF39*	*HAL9*	Y	Y	N	N	34%	filamentous	88
19.1718	*ZCF8*	ZN(2)-C6	Y	Y	N	Y	29%	148%	69
19.6781	*ZFU2*	*LYS14*	Y	Y	Y	Y	24%	20%	19
19.3187	*ZNC1*	*STB4*	Y	Y	N	N	29%	33%	22

Footnotes:

a These columns list each mutant according to the mutated gene (orf19 numbers and gene names).

b *S. cerevisiae* orthologs or best hits, or transcription factor classes, are indicated as listed in the Candida Genome Database.

c Column that indicates whether a deletion transcription factor mutant was available for adherence testing. All deletion mutants were created in the SN152 parent strain as described in Homann et al. 2009.

d Column that indicates whether a deletion transcription factor mutant was created and test for adherence. Strains were created in the BWP17 background and genotypes are in Table S4.

e These columns list the relative adherence for each mutant strain, and for each mutant strain derivative that carries the *ZAP1-OE* allele. The complete dataset for adherence measurements is in Table S1. All of the mutants and *ZAP1-OE* strains were insertion homozygotes except for *ace2, arg81, bcr1, crz2, snf5,* and *zfu2*, which were deletion homozygotes.

f This column lists the number of genes that were differentially expressed in each mutant compared to the wild-type control strain DAY185, as indicated by nanoString profiling. A cutoff p value of 0.05 was applied. Complete data and p values are in Table S2.

Cell-substrate adherence is often viewed as the first step in biofilm formation [1], [13]. Indeed, our findings above indicate that *BCR1* and *ACE2* are required for cell-substrate adherence, and prior studies have shown them to be required for biofilm formation [14], [15]. Therefore, all of the adherence-defective insertion mutants were tested for biofilm formation in vitro. Under our standard assay conditions [14], mutants defective in *SNF5* (discussed below) and *ARG81* (Figure S1) were unable to form adherent biofilms in vitro. Therefore, some adherence-defective mutants are defective in biofilm formation in vitro, while others represent a distinct functional class.

### Control of substrate adherence by Bcr1 and Als1

The transcription factor Bcr1 has been proposed to promote cell-cell adherence [16], but was not known to mediate cell-substrate adherence. We confirmed the substrate adherence defect of the *bcr1−/−* insertion mutant (Figure 1A) with the finding that a *bcr1Δ/Δ* deletion mutant had 3- to 4-fold reduced cell-substrate adherence compared to wild-type and complemented control strains (Figure 1B). (We refer to a homozygous insertion mutant as “*yfg1−/−*”, and a homozygous deletion mutant as “*yfg1Δ/Δ*”.) We tested the major known functional targets of Bcr1, which include adhesins Als1, Als3, and Hwp1 [16], [17], for roles in cell-substrate adherence. Deletion of *ALS1* alone caused a significant adherence defect, and overexpression of *ALS1* improved adherence in the *bcr1Δ/Δ* background (Figure 1B). Deletion of either *ALS3* or *HWP1* did not affect adherence (Figure 1B). These results indicate that Bcr1 is required for cell-substrate adherence, and that this function is mediated largely or entirely by the adhesin Als1.

### Roles of adherence regulators in gene expression

We used nanoString gene expression profiling to elucidate possible targets and pathway relationships among transcriptional regulators of adherence. RNA levels were measured for 293 genes. The surveyed genes included all 113 predicted GPI-linked cell surface protein genes [18], [19], representative gene targets of known biofilm regulators Ace2, Bcr1, and Zap1 [14], [15], [20], and a spectrum of genes related to hyphal formation, cell wall integrity, and stress responses (Table S2). We assayed gene expression in the 30 adherence-defective transcription factor mutants, five additional mutants with altered biofilm formation ability (*ire1−/−, gin4−/−, cbk1−/−, tec1−/−, zap1Δ/Δ* [14], [20], [21]), and the reference wild-type strain DAY185. Gene expression was assayed after growth for 8 hr at 37°C in liquid Spider medium, a medium we have used previously for analysis of biofilm-defective mutants [14], [20]. We used these growth conditions, despite the fact that they are different from those we used in our adherence assay, for two reasons. First, we sought to compare gene expression measurements with this new platform to our previously published microarray data. In fact, the new data agreed well with previous datasets: the nanoString probe set confirmed expression patterns for 20 previously reported Bcr1-regulated genes and 5 previously reported Zap1-regulated genes [14], [20]. Second, it seemed reasonable that gene expression comparisons among mutants might allow functional relationships to be inferred, regardless of the specific growth condition. Functional tests that we present below illustrate the value of the gene expression dataset for this purpose.

**Figure 2:**
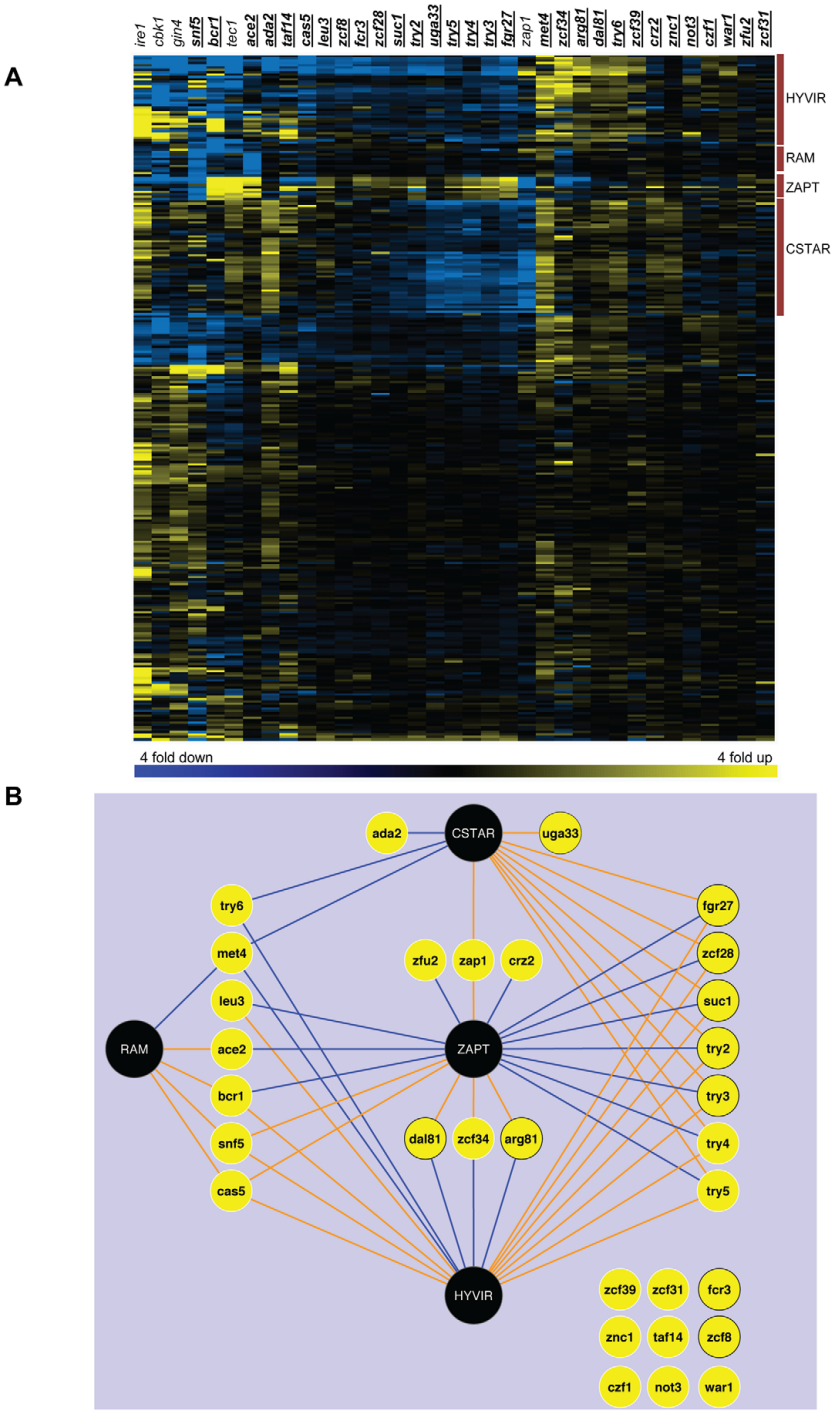
Gene expression profiles of adherence mutants. Panel A. Hierarchical clustering of gene expression data. NanoString expression data (Table S2) were analyzed as described in Methods. Briefly, averages of three independent determinations for each mutant strain were divided by averages of six independent determinations of the reference wild-type strain DAY185 to obtain the fold change values for each of 293 genes. All mutant strains were insertion homozygotes except for *ace2, arg81, crz2, zap1,* and *zfu2*, which were deletion homozygotes. Transcription factor mutants with adherence defects are indicated with underlined gene names; the remaining mutants were controls included for comparison. Color scale limits were set at (−2.0, 0.0, 2.0), so that the brightest yellow represents 4 fold up-regulation compared to wild-type, and the brightest blue represents 4 fold down-regulation. We define the clusters by representative genes. HYVIR: over 50% of the genes in this cluster are known to play roles in hyphal growth or virulence. RAM: top targets of Ace2 (Regulation of Ace2 and polarized morphogenesis), which are also regulated by Cbk1, Snf5, Cas5, Bcr1, and Met4. ZAPT: known Zap1 targets. CSTAR: Cell surface targets of adherence regulators. Additional small clusters of co-regulated genes did not have unifying functional or structural features. Panel B. Summary of regulatory relationships among the 30 adherence regulators, Zap1, and the four clusters of target genes defined in panel 2A. Black circles: target gene clusters. Yellow circles: transcription factors. Yellow circles with black border: adherence regulators whose defects in adherence can be rescued by *ZAP1* overexpression. Blue lines: negative regulation for at least 2/3 of the target genes in the cluster. Orange lines: positive regulation for at least 2/3 of the target genes in the cluster.

The adherence-defective mutants presented a range of pleiotropy in gene expression alterations (Table 1). Mutations in *WAR1, ZFU2,* and *ZNC1* had fairly mild effects, causing statistically significant changes in expression of only 16–22 of the genes assayed. Mutations in *ADA2, BCR1*, and *SNF5* were relatively severe, causing statistically significant changes in expression of 138–178 genes. Only two of the newly identified mutants had significantly reduced expression of *ALS1 (try3−/−* and *try4−/−*), and none had reduced expression of *BCR1,* thus indicating that the new mutations may define distinct adherence mechanisms (Table S2). An overview of the dataset reveals four striking findings (Figure 2A and 2B, Table S2). First, expression of a cluster of genes that includes hyphal- and virulence-associated genes (HYVIR cluster) is altered in 16 of the adherence-defective mutants. Interestingly, some additional genes (such as *CRH11, orf19.5626, HSP104*) cluster with the familiar hyphal/virulence genes, based on their co-regulation in several mutants, and may have previously unrecognized roles in these processes. Most of the mutants with altered hyphal/virulence gene expression have no previously described hyphal morphogenesis defect [12]. In the majority of these mutants, the hyphal/virulence genes are down-regulated compared to the wild type. Second, most targets of the transcription factor Ace2 (RAM cluster, named for “Regulation of Ace2 and polarized morphogenesis” [22]), are regulated by transcription factors Snf5, Cas5, Bcr1, and Met4. We probe the significance of the Snf5-RAM relationship below. Third, expression of zinc uptake genes and other known targets of the transcription factor Zap1 (ZAPT cluster [20]) is altered by 17 adherence-defective transcription factor mutants. For this set of genes, roughly equal numbers of mutants display up- or down-regulation. Finally, a novel group of 48 genes (CSTAR cluster [“Cell surface targets of adherence regulators”]) displays altered expression in 11 adherence-defective transcription factor mutants. The CSTAR genes include 37 genes that specify cell wall or secreted proteins. These genes are also regulated by the transcription factor Zap1; we examine the Zap1-adherence relationship below. There were additional clusters of co-regulated genes, but we could not define common functional or structural features among them. This overview has identified a major group of co-regulated genes, the CSTAR cluster, and has defined shared features among many of the new adherence regulators.

We used the gene expression data to deduce network relationships in order to define possible functional relationships among the adherence regulators (Figure 2B). This analysis points toward several findings. First, many adherence regulators control expression of two or more broad classes of target genes. For example, almost all regulators of the newly defined CSTAR genes also govern expression of HYVIR genes. Second, many groups of transcription factors have similar effects on their common target gene classes. For example, Fgr27, Zcf28, Suc1, Try2, Try3, Try4, and Try5 are all positive regulators of CSTAR and HYVIR genes, and negative regulators of ZAPT genes. Hence they may function together in a complex or pathway. Third, some transcription factors have opposite functions, such as Ada2 and Uga33 or Zfu2 and Zcf34. These relationships would be expected for one transcription factor that repressed expression of another, or for a repressor and an activator that recognize similar sequence motifs in front of target genes. Nine transcription factors (Fcr3, Zcf39, Zcf8, Zcf31, War1, Not3, Znc1, Taf14, and Czf1) did not have well defined target gene classes, and their possible relationships to other adherence regulators were not obvious. However, the profiling data do identify prospective target genes for all of these transcription factors (Figure 2A; Table S2) that may direct future studies. This network visualization suggests that many adherence regulators have common properties, and that many of these newly characterized transcription factors may converge to regulate a limited number of functional target genes or pathways.

### Functional relationship between Snf5 and Ace2

**Figure 3:**
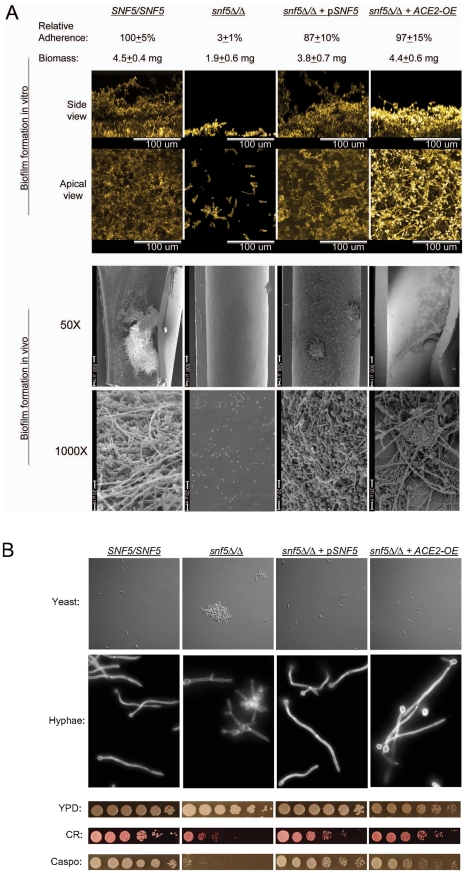
Functional relationship between Snf5 and Ace2. Strains indicated at the top of each column include *SNF5/SNF5* (DAY185), *snf5Δ/Δ* (DHY02), *snf5Δ/Δ+pSNF5* (DHY8), and *snf5Δ/Δ+ACE2-OE* (DHY20). Panel A. Adherence and biofilm formation assays. Each strain was assayed for adherence, biofilm formation in vitro (48 hr biomass measurements and 24 hr confocal imaging assays), and 24 hr biofilm formation in vivo (catheter lumen surfaces imaged via scanning electron microscopy at 50× or 1000× magnification as indicated). Panel B. Pleiotropic phenotypic assays. Yeast cells were visualized to assess aggregation after 8 hr growth (mid-exponential phase) in YPD at 30°C. Hypha formation visualized after 4 hr of growth in Spider medium at 37°C. Cell wall inhibitor sensitivity was measured by spot dilution assays: overnight cultures were serially diluted five-fold from left to right and assayed for growth on YPD, YPD+200 µg/ml Congo Red and YPD+62.5 µg/ml caspofungin after 48 hours at 30°C.

Snf5 is a subunit of the eukaryotic SWI/SNF chromatin remodeling complex [23], [24]. Both a *snf5Δ/Δ* deletion mutant and our original insertion mutant were defective in silicone adherence (Figure 1, Figure 3A). In addition, *snf5* mutants were defective in biofilm formation (Figure 3A). Confocal microscopic images showed sparse adherent cells, and mutant biofilms had diminished biomass. The *snf5* mutants also had pleiotropic phenotypic defects, including increased cell aggregation during yeast form growth, a severe defect in hyphal morphogenesis, and hypersensitivity to the cell wall inhibitors Congo Red and caspofungin (Figure 3B). Complementation of the *snf5Δ/Δ* mutant with a single copy of *SNF5* yielded phenotypes similar to the wild-type strain (Figure 3). These results indicate that loss of Snf5 function causes a spectrum of phenotypic defects.

The pleiotropic phenotypes of a *snf5Δ/Δ* mutant may be mediated by multiple regulatory pathways, in keeping with the global impact of the SWI/SNF complex on chromatin structure [25]. A second model, based on our gene expression analysis, is that many of the *snf5Δ/Δ* defects are the result of reduced *ACE2* expression. Although Ace2 is not known to govern cell wall integrity, it is known to affect adherence, biofilm formation, and hyphal morphogenesis [15], [22]. The second model predicts that many *snf5Δ/Δ* defects will be reversed by overexpression of *ACE2* in the mutant strain. To test that prediction, we fused the *TDH3* promoter to the *ACE2* coding region in the *snf5Δ/Δ* background, creating an *ACE2-OE* allele. Expression of *ACE2* was increased to approximately 3 times the wild type expression level, as indicated by QRTPCR assays (Figure S2). NanoString profiling confirmed that the *ACE2-OE* construct restored RAM gene expression in the *snf5Δ/Δ* mutant to nearly wild-type levels (preliminary results; Table S3). Overexpression of *ACE2* in the *snf5Δ/Δ* background restored adherence to wild-type levels (Figure 3A). In addition, it restored biofilm formation ability in vitro, as assayed by both biomass and confocal microscopic imaging (Figure 3A). Overexpression of *ACE2* caused substantial reversal of additional pleiotropic phenotypes, including yeast cell aggregation, hyphal morphogenesis, and sensitivity to cell wall inhibitors Congo Red and caspofungin (Figure 3B). These results indicate that much of the phenotypic impact of Snf5 stems from its role in *ACE2* expression.

To test the significance of our observations to infection, we turned to biofilm assays in vivo in a catheter infection model (Figure 3A). The *snf5Δ/Δ* mutant had a severe biofilm defect in vivo, and this defect was reversed by complementation with one wild-type copy of *SNF5*. Overexpression of *ACE2* partially restored biofilm formation in vivo as well. We conclude that *ACE2* is a pivotal Snf5 target gene that mediates multiple phenotypic properties, including biofilm formation in vitro and in vivo.

### Regulation of adherence by Zap1

Profiling data indicated that many adherence- and biofilm-defective mutants have altered expression of previously known Zap1-dependent genes (ZAPT genes in Figure 2). In addition, Zap1 is required along with several adherence regulators for expression of the newly described CSTAR genes. Given that a *zap1Δ/Δ* mutant has no detectable adherence defect (Figure 1A), we considered the hypothesis that Zap1 may act redundantly with another regulator or pathway to promote adherence. Our adherence-defective transcription factor mutants would likely include such a regulator. The hypothesis predicts that overexpression of *ZAP1* may improve adherence of mutants defective in the postulated redundant pathway.

**Figure 4:**
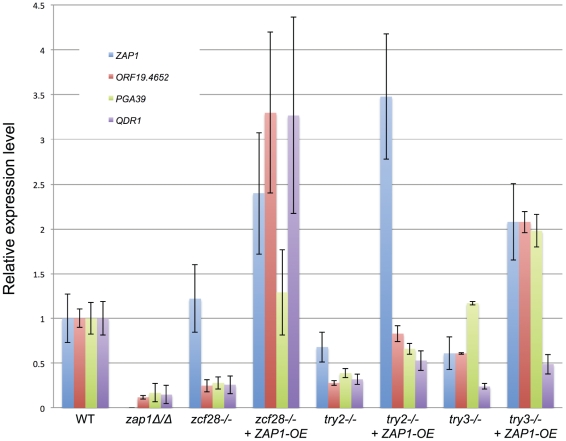
Expression of *ZAP1* and novel Zap1 dependent genes. Strains were grown in Spider medium for 8 hr at 37°C and QRTPCR assays were used to determine RNA levels for of *ZAP1, ORF19.4652, PGA39* and *QDR1.* RNA levels were normalized to control *TDH3* RNA and then expressed as relative units compared to each RNA in the wild-type strain. Strains included wild type (DAY185), *zap1Δ/Δ* (CJN1201), *zcf28Δ/Δ* (JF144), *zcf28Δ/Δ+ZAP1-OE* (JFY261), *try2−/−* (EHY97), *try2Δ/Δ+ZAP1-OE* (JFY337), *try3−/−* (EHY30), and *try3−/−+ZAP1-OE* (JFY251).

**Figure 5:**
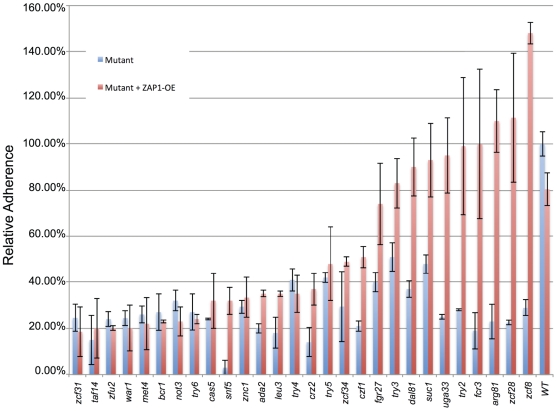
Restoration of adherence by increased *ZAP1* expression. Adherence of mutant strains, without the *ZAP1-OE* allele (blue) or with the *ZAP1-OE* allele (red), is indicated relative to the wild-type reference strain DAY185.

To test that prediction, we created derivatives of each transcription factor mutant that overexpress *ZAP1* from the *TDH3* promoter (*ZAP1-OE* allele). This allele resulted in 2- to 4-fold overexpression of *ZAP1* RNA in several representative mutants assayed (Figure 4). We confirmed the impact of *ZAP1* deletion and overexpression on target gene expression through QRTPCR assays (Figure 4). This analysis, conducted on three biological replicates, confirmed that three CSTAR genes were expressed at lower levels in the *zap1Δ/Δ* mutant than the wild-type strain (Figure 4). These three genes were also expressed at reduced levels in three adherence-defective mutants (*zcf28Δ/Δ, try2−/−,* and *try3−/−*), compared to the wild type. Importantly, expression of the three CSTAR genes increased when the *ZAP1-OE* allele was introduced into the mutants (Figure 4). These conclusions were extended with single nanoString determinations for several strains that were chosen on the basis of their adherence phenotypes presented below (preliminary results; Table S3). The *ZAP1-OE* construct increased CSTAR gene expression considerably in *arg81Δ/Δ, zcf28−/−, uga33−/−,* and *try2−/−* backgrounds (Table S3). In contrast, the *ZAP1-OE* construct had no effect on CSTAR gene expression in the *zcf34−/−* background. These observations suggest that *ZAP1* overexpression can stimulate CSTAR gene expression in some, but not all, adherence-defective mutants. We then compared adherence of each of the 30 mutant strains with and without the *ZAP1-OE* allele (Figure 5, Table 1). For ten mutants, the *ZAP1-OE* allele caused significantly increased adherence to a level comparable to the wild-type strain. This group included the *arg81Δ/Δ, zcf28−/−, uga33−/−,* and *try2−/−* mutants, in which *ZAP1-OE* caused increased CSTAR expression. The strains in which *ZAP1-OE* did not improve adherence included the *zcf34−/−* mutant, in which *ZAP1-OE* did not cause increased CSTAR gene expression. These findings argue that elevated expression of Zap1-dependent genes can alleviate the need for many transcription factors in promoting adherence.

## Discussion

**Figure 6:**
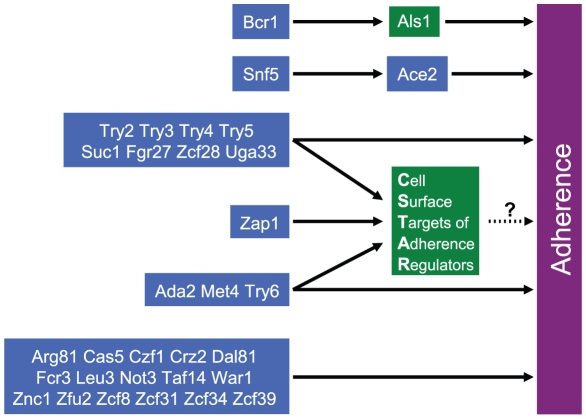
Portrait of *C. albicans* adherence regulators. Our main findings are summarized with transcription factors (blue boxes) connected to cell surface genes (green boxes) and the target process of adherence. Bcr1 promotes adherence through stimulation of *ALS1* expression. Snf5 promotes adherence through stimulation of *ACE2* expression. Try2, Try3, Try4, Try5, Suc1, Fgr27, Zcf28, and Uga33 are required for adherence and required for the expression of CSTAR genes. CSTAR gene products include numerous predicted cell wall proteins; we hypothesize that many CSTAR gene products mediate adherence. Zap1 is also a positive regulator of CSTAR genes, but it is not required for adherence in our assays. Ada2, Met4, and Try6 are negative regulators of many CSTAR genes. Finally, many transcription factors are required for adherence (Arg81, Cas5, Czf1, Crz2, Dal81, Fcr3, Leu3, Not3, Taf14, War1, Znc1, Zfu2, Zcf8, Zcf31, Zcf34, Zcf39), and govern expression of one or several classes of genes (summarized in Figure 2B), but cannot be connected to specific functional targets.

Adherence of *C. albicans* to a silicone substrate is critical for biofilm formation on implanted catheters, the basis for a major class of device-associated infections. Here we have viewed *C. albicans* adherence from the perspective of its transcriptional circuitry, using of a combination of mutant identification, gene expression profiling, and overexpression-rescue approaches. Our findings extend the detailed understanding of Bcr1 and Ace2, two transcription factors with previously described roles in biofilm formation (Figure 6). Our findings also have significant implications on a more global scale: they define a regulatory network through which twelve transcription factors govern expression of more than one quarter of the *C. albicans* cell surface protein genes (Figure 6). Among the twelve transcription factors is zinc response regulator Zap1, which also governs biofilm matrix accumulation and quorum sensing molecule production [20], [26]. Zap1 is thus positioned to coordinate multiple steps in biofilm formation. Finally, many adherence regulators do not have clear functional targets (Figure 6), based on our analysis. However, their unifying target gene classes (Figure 2B) will help to direct future studies.

### Cellular and regulatory functions of Bcr1

Bcr1 is among the best characterized biofilm regulators [1]. Previous studies indicated that its adhesin targets Als1/3 and Hwp1 mediate cell-cell interaction in biofilms [27]. Our findings extend that view by showing that Bcr1, through Als1, also governs cell-substrate adherence (Figure 6). Many previously known Bcr1 target genes are induced upon hyphal development [14], but *ALS1* is expressed in yeast form cells as well [28]. Although many Bcr1-dependent genes are hyphal genes, our findings here indicate that Bcr1 function in yeast form cells is biologically significant.

One striking feature to emerge from nanoString profiling is that Bcr1 governs expression of many more genes than any other transcription factor assayed except for chromatin remodeling factor Snf5 (Table 1). The set of genes assayed for expression was designed to include known Bcr1-dependent genes, so this result is not a fair measure of *bcr1Δ/Δ* mutant pleiotropy. However, our analysis of target gene clusters suggests that Bcr1 may be a constituent of the RAM network. Bcr1 has impact on hyphal morphogenesis [12], [14], like other RAM network components. In addition, we have recently found that Bcr1 is required for cell wall integrity (S. Fanning and A. P. Mitchell, unpublished results), a further parallel between Bcr1 and the RAM network. The mechanistic basis for interaction between Bcr1 and the RAM network is clearly an interesting area for further inquiry.

### Roles of Snf5 and Ace2 in biofilm formation

Our screen also revealed that Snf5, which functions in chromatin remodeling, is required for cell-substrate adherence. This finding in and of itself is not surprising, given that Snf5 is expected to govern expression of a multitude of different genes. What is striking is that such a broad spectrum of *snf5Δ/Δ* mutant phenotypes was reversed through increased expression of only one Snf5-dependent gene, *ACE2* (Figure 6). The relationship between Snf5 and Ace2 is clearly more intimate than previously appreciated, and an area that seems promising for more detailed mechanistic analysis.

Our analysis of the Snf5-Ace2 relationship suggests that a second transcription factor may be partially redundant with Ace2. Our logic is as follows. The *snf5Δ/Δ* mutant is hypersensitive to cell wall inhibitors, and overexpression of *ACE2* in the *snf5Δ/Δ* mutant reverses this hypersensitivity. This observation suggests that Ace2 promotes expression of genes that fortify the cell wall. Given that the *ace2Δ/Δ* mutant is not hypersensitive to cell wall inhibitors, then some other transcription factor may activate those genes in the absence of Ace2. Functional redundancy of Ace2 is consistent with a recent synthetic interaction study of the RAM network role in hyphal formation [29]. It is possible that Bcr1 is the Ace2-redundant transcription factor, because they share several target genes. In addition, overexpression of *BCR1* in the *snf5Δ/Δ* background relieves the mutant's caspofungin hypersensitivity (unpublished results). A second candidate is Cas5, a known regulator of cell wall integrity [30]. Our profiling data reveal that Cas5, like Bcr1, controls many RAM pathway genes. A third candidate is transcription factor Sko1, which is down-regulated in the *snf5Δ/Δ* mutant but not in the *ace2Δ/Δ* mutant (Table S2). Sko1 functions in *C. albicans* cell wall integrity [31]. Our profiling data provide only a small slice of what could be found through genome-wide analysis. However, the fact that our probe set focuses on known genes and pathways means that the results can be used efficiently to generate plausible hypotheses, as illustrated above.

### CSTAR cell surface network

Our results define a connection between 11 transcription factors that govern adherence, the zinc-response regulator Zap1, and 48 target genes that we refer to as CSTAR genes (Figure 2A). Among the CSTAR genes, 37 encode predicted surface or secreted proteins. Many of the predicted CSTAR products resemble adhesins, and three of them, Hwp2, Pbr1, and Pga10, have been shown to promote biofilm formation [32], [33]. A simple hypothesis is that one or several CSTAR gene products promote cell-substrate adherence (Figure 6).

Several preliminary results support a relationship between CSTAR gene products and adherence. One set of observations comes from nanoString profiling of *ZAP1-*overexpressing strains (see Table S3). In the *arg81Δ/Δ, try2−/−, uga33−/−,* and *zcf28−/−* backgrounds, *ZAP1* overexpression causes almost all CSTAR genes to reach or exceed their wild-type expression levels. In these strains, *ZAP1* overexpression rescues the adherence defect. Thus an increase in overall CSTAR gene expression levels correlates with increased adherence in these strains. In addition, we have found that most CSTAR genes are down-regulated in farnesol-treated biofilms (S. Ganguly, W. Xu, and A. P. Mitchell, unpublished results), a condition that promotes biofilm detachment [34]. Although these observations are preliminary, they are consistent with the model that one or several CSTAR gene products have a positive role in adherence.

Functional analysis of some CSTAR gene products suggests that their functions may be redundant [32], [33]. Specifically, deletions of CSTAR genes *HWP2, PBR1,* and *PGA10* cause only partial defects in adherence or biofilm formation [32], [33]. These findings imply that other gene products can compensate for absence of these three CSTAR genes to promote adherence [32], [33]. The analysis of Hwp2 indicates that it may have overlapping functions with Hwp1 and Rbt1 in promoting both mating and biofilm formation [32], [33]. We note that Hwp1 and Rbt1 are both hyphal genes and lie in our HYVIR cluster. Almost all adherence-defective transcription factor mutants with reduced CSTAR gene expression also have reduced HYVIR gene expression (see Figure 2B). An interesting possibility is that several CSTAR and HYVIR gene products make similar functional contributions to adherence.

Although we have considered the CSTAR genes as a single group, there are of course features that distinguish group members. For example, some CSTAR gene products are secreted (Sap1, Sap2, Sap3); some belong to protein families (Hyr3, Iff3, Iff4 [35]); some are transporters (Hgt12, Qdr1). In addition, some CSTAR genes are targets of only a subset of regulators: *PGA26* responds weakly to Zap1 and Zcf28; *IFF4* responds weakly to Suc1; *PGA46* and *CSA2* respond weakly to Met4 and Try6. These distinctions in regulation may reflect differences in transcription factor interactions or specificity, or perhaps overlapping regulatory networks that have compensatory effects. For example, Hap43 governs expression of 7 CSTAR genes [36]–[38], so Hap43 activity may influence the phenotypes of some CSTAR regulatory mutants. Similarly, the detailed spectrum of CSTAR expression alterations in any one adherence-defective mutant may affect its phenotype. We do not view the global analysis presented here as a substitute for more detailed analysis. Rather, this global portrait provides a basis for focusing detailed analysis, and a context in which to interpret it.

### Zap1 function and biofilm formation

Prior studies have shown that *C. albicans* Zap1 governs late events in biofilm formation, including production of extracellular matrix and quorum sensing molecules [20], [26]. Zap1 is required for efficient hypha formation under several conditions [12], [39], which is required for biofilm formation [1], [13]. Although the *zap1Δ/Δ* mutant has no adherence defect under our assay conditions, Zap1 is tied to adherence because its modest overexpression, in the range of 2- to 4-fold, restores adherence of 10 transcription factor mutants to a level comparable to the wild-type strain. We believe that this role is mediated through Zap1 control of CSTAR gene expression (Figure 6). The lack of an adherence defect for the *zap1Δ/Δ* mutant may reflect its ability to express some critical CSTAR genes, perhaps *PGA26* for example, or its ability to express potentially redundant HYVIR genes, as discussed above.

Both CSTAR genes and previously described Zap1 target (ZAPT) genes respond to mutations in many of the newly described adherence regulators. Surprisingly, these two sets of Zap1-dependent genes are not regulated in parallel. For example, the *ace2Δ/Δ, bcr1Δ/Δ, and zcf34−/−* strains have altered direct ZAPT gene expression but do not display altered CSTAR gene expression. Conversely, the *fgr27−/−, try3−/−, try4−/−, try5−/−,* and *uga33−/−* strains have reduced expression of many CSTAR genes, but have either no change or an increase in ZAPT gene expression. We cannot identify prospective Zap1 binding sites [20] in the 5′ regions of CSTAR genes, so they are probably regulated indirectly by Zap1. For example, Try4 or Try5 may be the direct activators of CSTAR genes; Try4/5 expression or activity may be stimulated by Zap1.

Zap1 target genes have been defined previously through microarray and ChIP-chip analyses [20]. However, CSTAR genes were not identified in that study. The previous analysis employed mature biofilm RNA, whereas here we have used planktonic RNA. However, we have verified that CSTAR genes are Zap1-dependent in mature biofilms as well (unpublished results). We believe that our detection of CSTAR gene expression differences reflects the fact that nanoString technology is much more sensitive than microarrays [11], and the CSTAR genes are expressed at low levels (roughly 1% of the level of *HWP1;* see Table S2). The identification of this novel class of target genes illustrates the well-known value of applying new technology to a scientific question.

### Adherence regulators and biofilm formation

Although we have identified numerous new adherence regulators, fairly few are required for biofilm formation in vitro. However, our preliminary results suggest that the assay is relevant to biofilm formation in vivo. Mutations in *ZFU2, CRZ2,* and *ZCF28* cause no biofilm defect in vitro, but block biofilm formation in the in vivo catheter model (unpublished results). It has not been feasible as of yet to test all 30 adherence defective mutants in vivo, but these results point to the validity of this approach to define genes relevant to infection.

## Materials and Methods

### Ethics statement

All procedures were approved by the Institutional Animal Care and Use Committee (IACUC) at the University of Wisconsin according to the guidelines of the Animal Welfare Act, The Institute of Laboratory Animal Resources Guide for the Care and Use of Laboratory Animals, and Public Health Service Policy.

### Strains and media

Strains were grown in yeast extract-peptone-dextrose (YPD) rich medium, Spider medium (1% nutrient broth (BD Difco), 1% D-mannitol (sigma), 0.2% K_2_HPO_4_ (Sigma)), or defined synthetic dextrose medium, prepared as previously described [26], [31], [40].

Unique strains used in this study are listed in Table S4. Insertion mutants were created as previously described [41]. The 197 UAU *his-* strains used in the initial adherence screen, as well as the transcription factor deletion mutants [12], are not listed here and are available at http://www.fgsc.net/candida/FGSCcandidaresources.htm. Deletion strains created in this study were made in the BWP17 background using PCR product-directed gene deletion as previously described [42]. Complementation of mutant strains was done as previously described [21]. Briefly, to complement a specific mutation, a fragment of DNA from ∼1000 bp upstream to ∼300 bp downstream of an open reading frame was amplified from BWP17 genomic DNA. Primers contained a 40 bp sequence added to the 5′ end to allow in vivo recombination into plasmid pSG1. The plasmid pSG1 was derived by replacing the *URA3-f1-lacZ* sequence from the vector pRS416 with the *C. albicans HIS1* including a NruI restriction site [43]. The amplified PCR fragment and NotI linearized pSG1 was co-transformed into *S. cerevisiae* strain AMP271 with the resulting plasmid amplified in *E. coli*. The complementation plasmid was then digested with NruI and transformed into the respective mutant strain to target insertion to the *HIS1* locus. All complementation was confirmed by QRT-PCR as previously described [21]. Primers used to create the deletions and the complemented strains are listed in Table S5.

Creation of EHY strains were accomplished by standard *C. albicans* transformation protocols [44]. The specific CJN, FJS, DSY and SFY strains were transformed with NruI digested plasmid pDDB78 [45], and selected on synthetic dextrose medium lacking histidine. Isolates were streaked for singles and 3 independent *HIS+* UAU insertion isolates were confirmed by PCR.

Overexpression of *ZAP1* in the 30 adherence defective mutants was accomplished by replacing the endogenous *ZAP1* promoter (at one allele) with the promoter of *TDH3* as described previously [20]. For *ZAP1* overexpression, primers pTDH3 ZAP1 FOR, and pTDH3 ZAP1 REV, were used to amplify the *THD3* promoter, with the resulting PCR product being used for recombination into *ZAP1* promoter.

For complementation of mutant strains, PCR primers were designed to amplify genomic DNA of strain SC5314 from 1 kb upstream to 0.5 kb downstream of the open reading frame of a specific gene. Shorter distances were used when there were additional genes located within this region. The resulting PCR product was cotransformed into *S. cerevisiae* with EcoRI and NotI digested plasmid pDDB78. Plasmid DNA was isolated, transformed into *E. coli*, and isolated plasmid DNA was digested with NruI and transformed into the respective *C. albicans* mutant strains. Presence of the relevant insertion mutation was verified by genomic PCR using internal and flanking primers.

New gene names were assigned as follows. The *S. cerevisiae* ortholog of *orf19.5871* is *ScSNF5,* so we use the name *SNF5* for *orf19.5871*. Other previously unnamed genes are designated *TRY* genes (Transcriptional Regulators of Yeast cell adherence); we refer to *orf19.4062* as *TRY2, orf19.1971* as *TRY3, orf19.5975* as *TRY4*, *orf19.3434* as *TRY5,* and *orf19.6824* as *TRY6.* We had initially referred to *orf19.6781* as *TRY1*, but the name *ZFU2* was posted at the *Candida* Genome Database during the course of our studies.

### Cell wall sensitivity assays

Strains were tested for drug sensitivity as described previously [30]. Briefly, overnight cultures in YPD were diluted to an OD_600_ of 3.0 and serially diluted five-fold and spotted onto YPD, YPD plus 62.5 µg/ml of caspofungin, and YPD plus 200 µg/ml Congo red plates. Plates were incubated at 30°C for 24–48 hours.

### Yeast and hyphal growth assays

Yeast cell morphology was assayed as previously described [40]. Briefly, overnight cultures grown at 30°C in liquid YPD were diluted to an OD_600_ of 0.2 with fresh YPD medium and were grown at 30°C to an OD_600_ of ∼0.8. Cells were visualized using a Zeiss Axio Observer Z.1 microscope with a 20× NA 1.4 objective. Digital photographs were acquired on a Coolsnap HQ^2^ (Photometrics) camera using Axiovision (Zeiss) software. For hyphal growth assay, overnight cultures grown at 30°C in liquid YPD were diluted to an OD_600_ of 0.08 in Spider medium. Cultures were agitated at 220-rpm at 37°C for 180 minutes. The samples were then washed with phosphate-buffered saline (PBS), and incubated for ten minutes in PBS+0.125 mg/ml calcofluor white (Sigma) [46]. Cells were visualized as described above but with a 63× objective. ImageJ was used to process the images.

### In vitro biofilm assays

Biofilm formation assays were performed as previously described [14]. Briefly, overnight cultures grown at 30°C in liquid YPD were diluted to OD_600_ of 0.5 in 2 ml of Spider medium, and incubated with silicone squares coated with fetal bovine serum. After 90 min incubation at 37°C with 70-rpm agitation, the silicone squares were washed with 2 ml PBS to remove any unadhered cells, and 2 ml of fresh Spider medium were added. After 48 hr incubation at 37°C with 70-rpm agitation the silicone squares were photographed and analyzed for biofilm growth.

Biofilm dry masses were performed as previously described [40]. Briefly, biofilms were grown on silicone squares for 48 hours. Silicone squares were vortexed in ddH_2_O to completely detach the cells from the silicone surface. The cells were collected under suction on pre-weighed 0.45 µm nitrocellulose filters (Millipore). After four days of drying the filters were weighed. For each strain the measurement was in triplicate.

### In vitro biofilm visualization by confocal microscopy

Biofilms were grown as described above, except that the incubation period was 24 hours. After 24 hours growth, biofilms were gently washed with 2 ml of 1× PBS. The biofilm was then incubated in 2 ml of 1× PBS with Calcofluor stain at a final concentration of 0.125 mg/ml for 10 minutes at 37°C with agitation at 70-rpm [46]. A 60 mm dish (Fisher) was punched with a 17×17 mm square hole and a No. 1 glass coverslip was fused to the bottom of the dish with UV-curing cement (Norland NOA-61) to form a shallow well. Double-sided tape was attached to the interior glass bottom of the well, to act as a spacer preventing contact of the inverted biofilm to the bottom of the dish. Once the dish was completed, 300 µl PBS+calcofluor solution was added to the well. The biofilm was then carefully removed and inverted, and gently placed onto the double sided tape. After the biofilm was inverted and affixed to the coverslip, 7 ml of PBS/calcofluor solution was added to the dish. The biofilm was then imaged with a Zeiss LSM 510 Meta/DuoScan inverted spectral confocal microscope using a 40× water immersion 1.2 NA objective with the laser line at 405 nm. The Zen 2009 software was used to obtain the desired Z stack images. Image J (http://rsbweb.nih.gov/ij/) was used to create the side view image and apical view.

### In vivo biofilm model

A rat central-venous-catheter infection model was used to assay in vivo biofilms, as previously described [47]. Briefly, after 24 hours of *C. albicans* infection catheters are removed from the rat and the distal 2 cm of catheter material is removed and assayed for biofilm growth via imaging using scanning electron microscopy (SEM) [48].

### Yeast cell adherence assay

Adherence assays were conducted with Fluxion BioFlux 200, a flow apparatus with micron scale fluidic channels that allows visualization of adherent cells with controlled flow rates. The flow chamber consists of a glass coverslip plasma fused to the fluidic channel constructed out of polydimethylsiloxane. *C. albicans* cells bind to the polydimethylsiloxane but not to the glass.

Strains of interest were grown overnight in YPD at 30°C, and agitated at 220-rpm. The strains were diluted to an OD_600_ of 0.2 in YPD medium. 500 µl of sample was added to each lane and each sample was run in duplicate. For each plate a reference strain was run, which later was used for fold comparison to the mutant. For the *his-* strains the reference strain was DAY286, for EHY *HIS+* strains the reference strain was DAY185, and for the Homann collection the reference strain was SN250 [12]. After loading, a flow rate of 3 dyn/cm^3^ was applied for 30 minutes at 30°C. After 30 minutes of flow each lane had two images taken at different sites along the channel. Images were always taken at the same location in each channel for each sample. Strains with filamentous or clumping cells were not assayable. For each image the number of yeast cells adhering to the channel was tabulated. Since two pictures were taken per lane the sum of each lane was used as a single determinate and each strain thus had two trials. The average was taken for each strain and the fold change calculated (number of yeast cells adhered in the mutant strain/number of yeast cells adhered in the reference strain). Error bars were calculated by standard deviation, p-values were calculated by t-test. For strains that had significant changes in adherence, a second isolate of the strain was assayed to confirm the initial results.

### Quantitative RTPCR

10 µg of isolated RNA was DNase treated (Ambion), and AffinityScript multiple temperature cDNA synthesis kit (Stratagene) was used for first-strand cDNA synthesis. A control reaction lacking the reverse transcriptase was performed to ensure absence of DNA contamination. Quantification was performed for gene amplification for the gene of interest and the reference, *TDH3*. All data was normalized to *TDH3*. Primers used for PCR amplification are listed in Table S5. QRTPCR reactions were prepared and performed on a Biorad iQ5 as previously described [21].

### NanoString probe choice

One key issue with nanoString determination is to choose informative genes for expression measurements. We have chosen 300 genes for assays, based on four considerations. First, we included probes for ∼150 known or predicted cell wall genes, including 113 genes with potential GPI lipid modification sites identified by an earlier study [19]. Second, we included probes for ∼50 genes known to play a role in host-pathogen interactions, such as the *ALS* and *SAP* gene families. Third, based on previous genome wide expression studies [30], [41], we included probes for ∼100 genes that are highly regulated during hypha development or biofilm formation, during cell wall stress, and in osmotic or oxidative stress conditions. Fourth, we included control genes for high, moderate and low expression classes. We chose two genes of each class that vary little in numerous microarray studies from our lab as internal controls. They are *ACT1, TDH3* (high), *ARP3, orf19.5917.3* (moderate), *orf19.7235, PTC1* (low). The list of genes and their orf19 numbers are shown in Table S2.

### NanoString sample preparation and data collection

Cells from overnight YPD cultures were inoculated into 50 ml Spider medium at OD 0.2, and were grown for 8 hours at 37°C with 220-rpm agitation before harvesting. Cells were collected by filtering through 0.45 um nitrocellulose filters (Millipore). Half of each culture (25 ml) was collected on one filter paper and let dry at room temp for dry weight measurements, the other half was collected on a separate filter and immediately frozen at −80°C for RNA extraction. Total RNA was extracted using the Qiagen RNeasy Plant kit (Cat #74904). 80 ng of total RNA was mixed with the nanoString probe set and incubated at 65°C overnight (12–18 hours). The reaction mix was then loaded on the nanoString nCounter Prep Station for binding and washing, using the default program. The resultant cartridge was then transferred to the nanoString nCounter digital analyzer for scanning and data collection. A total of 600 fields were captured per sample. Three independent samples were prepared and processed for each mutant (six samples for the wildtype control strain DAY185). We performed nanoString analysis on 30 transcription factor mutants with reduced yeast form adherence, 2 transcription factor mutant strains (*tec1*, *zap1*) that have wild-type levels of adherence, and 3 protein kinase mutant strains that are known to have severe defects in cell wall integrity and biofilm formation (*ire1, gin4* and *cbk1*) [21]. All 35 mutants are listed in Table S2.

### NanoString data analysis

The raw data, in a form of digital counts for each of the 300 genes in every sample, were first adjusted for binding efficiency and background subtraction using the manufacturer included positive and negative controls, following nCounter data analysis guidelines. Second, mutant strain data sets were normalized to the control wildtype strain DAY185 using three groups of control genes: *ACT1, TDH3* (high), *ARP3, orf19.5917.3* (moderate), *orf19.7235, PTC1* (low). Normalization factors were calculated for each group, and the average of the three was used to normalize the whole data set. We noticed that the normalization factors calculated for the three groups (high, moderate and low) were very consistent, usually within 10% difference. The normalized data sets for 35 mutants, each containing expression data for 293 genes, were shown in Table S2 and were further analyzed (we took out the 6 control genes, and *OSM1*, which is the same as *ALS4*. *OSM1* was annotated as a separate gene adjacent to *ALS4*, but was later corrected as a part of the *ALS4* gene. Our readings on *OSM1* and *ALS4* were almost identical in all mutants and wildtype).

We used MultiExperimentViewer (MeV v4.6.2) to cluster the data sets. The normalized data sets were used to determine if the expression level of a gene in a mutant was significantly different from that in the wild-type control by two-tailed Student t-test. For ones that are significantly different (P<0.05), the average of three determinations for a gene in a mutant was divided by the average of six determinations for the respective gene in the wild-type control to calculate the fold change. For ones that are not significantly different (P>0.05), we set the fold change as 1, so that they would not affect clustering analysis. The data (fold changes comparing to wildtype) were log2 transformed, and hierarchical clustered by averaging linkage clustering based on Manhattan Distance, and optimized for gene leaf order. Color scale limits were set at “−2.0, 0.0, 2.0”, meaning that the brightest yellow represents 4 fold upregulation comparing to wild-type, the brightest blue represents 4 fold downregulation, and black represents no change (or the change is not considered significant by t-test). We also performed the same clustering analysis using the original normalized data sets (i.e. without using the t-test to eliminate ones with p-value >0.05). The resultant clusters were very similar to what we obtained using the p-value adjusted data sets. The clustering diagram shown in figure 2A is from the original data sets.

## Supporting Information

Figure S1 Biofilm formation assays of *ARG81/ARG81, arg81Δ/Δ,* and *arg81Δ/Δ+pARG81* strains. Biofilm formation was assayed in vitro for 48 hr.
(PPT)

Figure S2 RNA Levels of *SNF5* and *ACE2* in strains *SNF5/SNF5*, *snf5Δ/Δ, snf5Δ/Δ+pSNF5,* and *snf5Δ/Δ+ACE2-OE* strains. RNA levels were measured by QRTPCR and normalized to control *TDH3* RNA levels.
(PPT)

Table S1 Complete adherence measurements for mutant and complemented strains. Adherence determinations were made with a Fluxion flow cell, and are listed as mean and standard deviation.
(XLS)

Table S2 NanoString expression data for mutant strains. NanoString measurements of reporter gene expression are provided as raw numbers for individual assays, as well as means and standard deviations.
(XLS)

Table S3 NanoString expression data for *ACE2-OE* and *ZAP1-OE* strains. NanoString measurements of reporter gene expression are provided as raw numbers for individual assays, as well as means and standard deviations.
(XLS)

Table S4 Genotypes of *C. albicans* strains. Complete genotypes of *C. albicans* strains used in this study are listed.
(DOC)

Table S5 Oligonucleotide sequences. Sequences of oligonucleotides used in this study are indicated, exclusive of nanoString probes.
(XLSX)
