# Supplementary material for: Portrait of Candida albicans Adherence Regulators
Source: PLoS Pathog. 2012 Feb 16;8(2):e1002525. doi: 10.1371/journal.ppat.1002525 (PMC3280983; doi:10.1371/journal.ppat.1002525)
Supplement: Figure S1 — Biofilm formation assays of ARG81/ARG81, arg81Δ/Δ, and arg81Δ/Δ+pARG81 strains. Biofilm formation was assayed in vitro for 48 hr. (PPT) [file ppat.1002525.s001.ppt]

## Slide 1
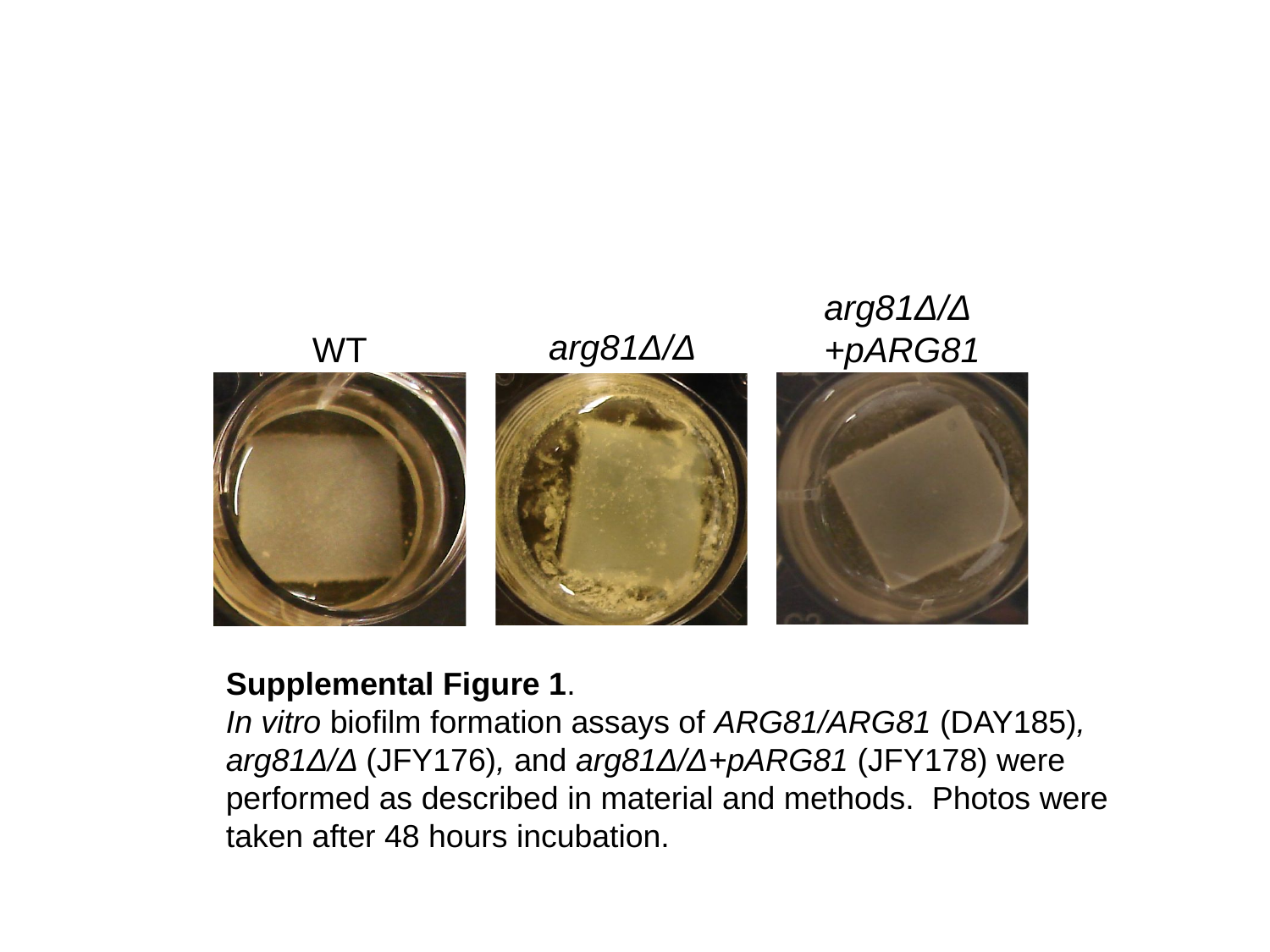

arg81Δ/Δ
+pARG81
arg81Δ/Δ
WT
Supplemental Figure 1.
In vitro biofilm formation assays of ARG81/ARG81 (DAY185), arg81Δ/Δ (JFY176), and arg81Δ/Δ+pARG81 (JFY178) were performed as described in material and methods. Photos were taken after 48 hours incubation.
