# Supplementary material for: Portrait of Candida albicans Adherence Regulators
Source: PLoS Pathog. 2012 Feb 16;8(2):e1002525. doi: 10.1371/journal.ppat.1002525 (PMC3280983; doi:10.1371/journal.ppat.1002525)
Supplement: Figure S2 — RNA Levels of SNF5 and ACE2 in strains SNF5/SNF5, snf5Δ/Δ, snf5Δ/Δ+pSNF5, and snf5Δ/Δ+ACE2-OE strains. RNA levels were measured by QRTPCR and normalized to control TDH3 RNA levels. (PPT) [file ppat.1002525.s002.ppt]

## Slide 1
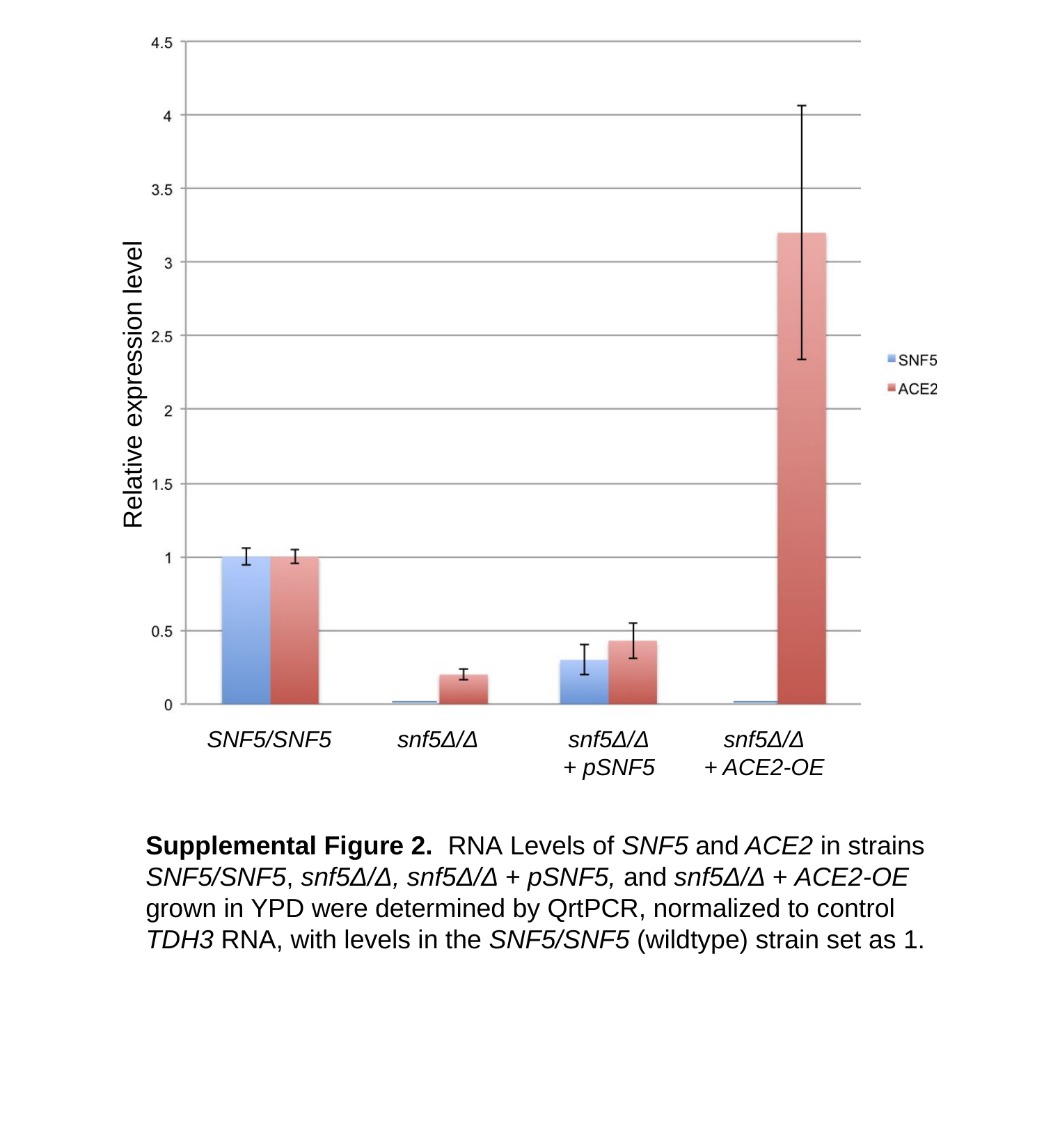

Relative expression level
SNF5/SNF5
snf5Δ/Δ
snf5Δ/Δ
+ pSNF5
snf5Δ/Δ
+ ACE2-OE
Supplemental Figure 2. RNA Levels of SNF5 and ACE2 in strains SNF5/SNF5, snf5Δ/Δ, snf5Δ/Δ + pSNF5, and snf5Δ/Δ + ACE2-OE grown in YPD were determined by QrtPCR, normalized to control TDH3 RNA, with levels in the SNF5/SNF5 (wildtype) strain set as 1.
