# Supplementary material for: Portrait of Candida albicans Adherence Regulators
Source: PLoS Pathog. 2012 Feb 16;8(2):e1002525. doi: 10.1371/journal.ppat.1002525 (PMC3280983; doi:10.1371/journal.ppat.1002525)
Supplement: Table S4 — Genotypes of C. albicans strains. Complete genotypes of C. albicans strains used in this study are listed. (DOC) [file ppat.1002525.s006.doc]

| Strain name | Genotype | Source | Reference |
| --- | --- | --- | --- |
| BWP17 | *ura3∆::λimm434 arg4::hisG his1::hisG*  *ura3∆::λimm434 arg4::hisG his1::hisG* | RM1000 | {Wilson, 1999 #2} |
| DAY185 | *ura3∆::λimm434 ARG4:URA3:arg4::hisG his1::hisG::pHIS1*  *ura3∆::λimm434 arg4::hisG his1::hisG:* | BWP17 | {Davis, 2000 #54} |
| DAY286 | *ura3∆::λimm434 ARG4:URA3:arg4::hisG his1::hisG*  *ura3∆::λimm434 arg4::hisG his1::hisG:* | BWP17 | {Davis, 2000 #55} |
| CJN698 | ura3∆::λimm434 arg4::hisG his1::hisG::pHIS1-BCR1 bcr1::ARG4ura3∆::λimm434 arg4::hisG his1::hisG bcr1::URA3 | BWP17 | {Nobile, 2005 #14} |
| CJN702 | ura3∆::λimm434 arg4::hisG his1::hisG::pHIS1 bcr1::ARG4ura3∆::λimm434 arg4::hisG his1::hisG bcr1::URA3 | BWP17 | {Nobile, 2005 #14} |
| CJN896 | ura3∆::λimm434 arg4::hisG his1::hisG::pHIS1 tec1::Tn7-UAU1ura3∆::λimm434 arg4::hisG his1::hisG tec1::Tn7-URA3 | BWP17 | {Nobile, 2005 #14} |
| CJN1144 | ura3∆::λimm434 arg4::hisG his1::hisG::pHIS1 bcr1::ARG4 TEF1-ALS1::NAT1 *ura3∆::λimm434 arg4::hisG his1::hisG bcr1::URA3 ALS1* | BWP17 | {Nobile, 2006 #48} |
| CJN1201 | ura3∆::λimm434 arg4::hisG his1::hisG::pHIS1 zap1::ARG4ura3∆::λimm434 arg4::hisG his1::hisG zap1::URA3 | BWP17 | {Nobile, 2009 #30} |
| CJN1659 | ura3∆::λimm434 ARG4:URA3::arg4::hisG his1::hisG::pHIS1 YWP1:::pAgTEF1-NAT1-AgTEF1UTR-TDH3-YWP1ura3∆::λimm434 arg4::hisG his1::hisG YWP1 | DAY185 | {Nobile, 2009 #30} |
| CAYF178U | *ura3∆::λimm434::URA3-IRO1 als3::ARG4 arg4::hisG his1::hisG* ura3∆::λimm434 als3::HIS1 arg4::hisG his1::hisG | CAI-4 | {Nobile, 2006 #48} |
| CAYC1 | *ura3∆::λimm434::URA3-IRO1 als1::hisG::ALS1* ura3∆::λimm434 als1::hisG | CAI-4 | {Fu, 2002 #61} |
| CAYC2YF1U | *ura3∆::λimm434::URA3-IRO1 als1::hisG* ura3∆::λimm434 als1::hisG: | CAI-4 | {Fu, 2002 #61} |
| CAH7-1A1E2 | *ura3∆::λimm434 hwp1::hisG eno1::URA2* ura3∆::λimm434 hwp1::hisG ENO1 | CAI-4 | {Staab, 1999 #62} |
| DAY951 | ura3∆::λimm434 arg4::hisG his1::hisG::pHIS1 crz2::ARG4ura3∆::λimm434 arg4::hisG his1::hisG crz2::URA3-dpl200 | BWP17 | {Kullas, 2007 #53} |
| DAY954 | ura3∆::λimm434 arg4::hisG his1::hisG::pHIS1-CRZ2 crz2::ARG4ura3∆::λimm434 arg4::hisG his1::hisG crz2::URA3-dpl200 | BWP17 | {Kullas, 2007 #53} |
| JJH276 | ura3∆::λimm434 arg4::hisG his1::hisG::pHIS1 gin4::Tn7-UAU1ura3∆::λimm434 arg4::hisG his1::hisG gin4::Tn7-URA3 | BWP17 | {Blankenship, 2010 #26} |
| CTN85 | ura3∆::λimm434 arg4::hisG his1::hisG::pHIS1 ace2::ARG4ura3∆::λimm434 arg4::hisG his1::hisG ace2::URA3 | BWP17 | This study |
| CTN90 | ura3∆::λimm434 arg4::hisG his1::hisG::pHIS1-ACE2 ace2::ARG4ura3∆::λimm434 arg4::hisG his1::hisG ace2::URA3 | BWP17 | This study |
| EHY14 | ura3∆::λimm434 arg4::hisG his1::hisG::pHIS1 ada2::Tn7-UAU1ura3∆::λimm434 arg4::hisG his1::hisG ada2::Tn7-URA3 | CJN863 | This study |
| EHY15 | ura3∆::λimm434 arg4::hisG his1::hisG::pHIS1 ada2::Tn7-UAU1ura3∆::λimm434 arg4::hisG his1::hisG ada2::Tn7-URA3 | CJN863 | This study |
| EHY24 | ura3∆::λimm434 arg4::hisG his1::hisG::pHIS1 cas5::Tn7-UAU1ura3∆::λimm434 arg4::hisG his1::hisG cas5::Tn7-URA3 | CJN432 | This study |
| EHY25 | ura3∆::λimm434 arg4::hisG his1::hisG::pHIS1 cas5::Tn7-UAU1 *ura3∆::λimm434 arg4::hisG his1::hisG cas5::Tn7-URA3* | CJN432 | This study |
| EHY26 | ura3∆::λimm434 arg4::hisG his1::hisG::pHIS1 try4::Tn7-UAU1 *ura3∆::λimm434 arg4::hisG his1::hisG try4::Tn7-URA3* | CJN809 | This study |
| EHY27 | ura3∆::λimm434 arg4::hisG his1::hisG::pHIS1 try4::Tn7-UAU1 *ura3∆::λimm434 arg4::hisG his1::hisG try4::Tn7-URA3* | CJN809 | This study |
| EHY30 | ura3∆::λimm434 arg4::hisG his1::hisG::pHIS1 try3::Tn7-UAU1 *ura3∆::λimm434 arg4::hisG his1::hisG try3::Tn7-URA3* | SFY42 | This study |
| EHY34 | ura3∆::λimm434 arg4::hisG his1::hisG::pHIS1 znc1::Tn7-UAU1 *ura3∆::λimm434 arg4::hisG his1::hisG znc1::Tn7-URA3* | SFY42 | This study |
| EHY39 | ura3∆::λimm434 arg4::hisG his1::hisG::pHIS1 fgr27::Tn7-UAU1 *ura3∆::λimm434 arg4::hisG his1::hisG fgr27::Tn7-URA3* | CJN419 | This study |
| EHY40 | ura3∆::λimm434 arg4::hisG his1::hisG::pHIS1 fgr27::Tn7-UAU1 *ura3∆::λimm434 arg4::hisG his1::hisG fgr27::Tn7-URA3* | CJN419 | This study |
| EHY43 | ura3∆::λimm434 arg4::hisG his1::hisG::pHIS1 zcf39::Tn7-UAU1 *ura3∆::λimm434 arg4::hisG his1::hisG zcf39::Tn7-URA3* | CJN495 | This study |
| EHY46 | ura3∆::λimm434 arg4::hisG his1::hisG::pHIS1 czf1::Tn7-UAU1 *ura3∆::λimm434 arg4::hisG his1::hisG czf1::Tn7-URA3* | CJN517 | This study |
| EHY54 | ura3∆::λimm434 arg4::hisG his1::hisG::pHIS1 taf14::Tn7-UAU1 *ura3∆::λimm434 arg4::hisG his1::hisG taf14::Tn7-URA3* | CJN856 | This study |
| EHY55 | ura3∆::λimm434 arg4::hisG his1::hisG::pHIS1 taf14::Tn7-UAU1 *ura3∆::λimm434 arg4::hisG his1::hisG taf14::Tn7-URA3* | CJN856 | This study |
| EHY60 | ura3∆::λimm434 arg4::hisG his1::hisG::pHIS1 fcr3::Tn7-UAU1 *ura3∆::λimm434 arg4::hisG his1::hisG fcr3::Tn7-URA3* | CJN926 | This study |
| EHY61 | ura3∆::λimm434 arg4::hisG his1::hisG::pHIS1 fcr3::Tn7-UAU1 *ura3∆::λimm434 arg4::hisG his1::hisG fcr3::Tn7-URA3* | CJN926 | This study |
| EHY67 | ura3∆::λimm434 arg4::hisG his1::hisG::pHIS1 dal81::Tn7-UAU1 *ura3∆::λimm434 arg4::hisG his1::hisG dal81::Tn7-URA3* | DSY3414-1 | This study |
| EHY70 | ura3∆::λimm434 arg4::hisG his1::hisG::pHIS1 zcf31::Tn7-UAU1 *ura3∆::λimm434 arg4::hisG his1::hisG zcf31::Tn7-URA3* | DSY3420-1 | This study |
| EHY73 | ura3∆::λimm434 arg4::hisG his1::hisG::pHIS1 war1::Tn7-UAU1 *ura3∆::λimm434 arg4::hisG his1::hisG war1::Tn7-URA3* | DSY3429-1 | This study |
| EHY75 | ura3∆::λimm434 arg4::hisG his1::hisG::pHIS1 zcf8::Tn7-UAU1 *ura3∆::λimm434 arg4::hisG his1::hisG zcf8::Tn7-URA3* | DSY3447-11 | This study |
| EHY76 | ura3∆::λimm434 arg4::hisG his1::hisG::pHIS1 zcf8::Tn7-UAU1 *ura3∆::λimm434 arg4::hisG his1::hisG zcf8::Tn7-URA3* | DSY3447-11 | This study |
| EHY78 | ura3∆::λimm434 arg4::hisG his1::hisG::pHIS1 not3::Tn7-UAU1 *ura3∆::λimm434 arg4::hisG his1::hisG not3::Tn7-URA3* | HL11-1 | This study |
| EHY79 | *ura3∆::λimm434 arg4::hisG his1::hisG::pHIS1 not3::Tn7-UAU1* ura3∆::λimm434 arg4::hisG his1::hisG not3::Tn7-URA3 | HL11-1 | This study |
| EHY85 | ura3∆::λimm434 arg4::hisG his1::hisG::pHIS1 suc1::Tn7-UAU1 *ura3∆::λimm434 arg4::hisG his1::hisG suc1::Tn7-URA3* | RLS3 | This study |
| EHY87 | ura3∆::λimm434 arg4::hisG his1::hisG::pHIS1 leu3::Tn7-UAU1 *ura3∆::λimm434 arg4::hisG his1::hisG leu3::Tn7-URA3* | RLS18 | This study |
| EHY88 | *ura3∆::λimm434 arg4::hisG his1::hisG::pHIS1 leu3::Tn7-UAU1*  *ura3∆::λimm434 arg4::hisG his1::hisG leu3::Tn7-URA3* | RLS18 | This study |
| EHY90 | ura3∆::λimm434 arg4::hisG his1::hisG::pHIS1 try5::Tn7-UAU1 *ura3∆::λimm434 arg4::hisG his1::hisG try5:Tn7-URA3* | RLS50 | This study |
| EHY91 | *ura3∆::λimm434 arg4::hisG his1::hisG::pHIS1 try5::Tn7-UAU1* ura3∆::λimm434 arg4::hisG his1::hisG try5::Tn7-URA3 | RLS50 | This study |
| EHY97 | ura3∆::λimm434 arg4::hisG his1::hisG::pHIS1 try2::Tn7-UAU1 *ura3∆::λimm434 arg4::hisG his1::hisG try2::Tn7-URA3* | SFY7 | This study |
| EHY103 | ura3∆::λimm434 arg4::hisG his1::hisG::pHIS1 try6::Tn7-UAU1 *ura3∆::λimm434 arg4::hisG his1::hisG try6::Tn7-URA3* | SFY10 | This study |
| EHY106 | ura3∆::λimm434 arg4::hisG his1::hisG::pHIS1 met4::Tn7-UAU1ura3∆::λimm434 arg4::hisG his1::hisG met4:Tn7-URA3 | SFY39 | This study |
| EHY108 | ura3∆::λimm434 arg4::hisG his1::hisG::pHIS1 zcf34::Tn7-UAU1 *ura3∆::λimm434 arg4::hisG his1::hisG zcf34::Tn7-URA3* | CJN548 | This study |
| EHY119 | ura3∆::λimm434 arg4::hisG his1::hisG::pHIS1 cbk1::Tn7-UAU1ura3∆::λimm434 arg4::hisG his1::hisG cbk1::Tn7-URA3 | JJH113 | This study |
| JFY144 | ura3∆::λimm434 arg4::hisG his1::hisG::pHIS1 zcf28::ARG4 *ura3∆::λimm434 arg4::hisG his1::hisG zcf28::URA3* | BWP17 | This study |
| JFY146 | ura3∆::λimm434 arg4::hisG his1::hisG::pHIS1 arg81::Tn7-UAU1ura3∆::λimm434 arg4::hisG his1::hisG arg81::Tn7-URA3 | CJN401 | This study |
| JFY150 | ura3∆::λimm434 arg4::hisG his1::hisG::pHIS1-ZFU2 zfu2::ARG4ura3∆::λimm434 arg4::hisG his1::hisG zfu2::URA3 | BWP17 | This study |
| JFY151 | ura3∆::λimm434 arg4::hisG his1::hisG::pHIS1 zfu2::ARG4ura3∆::λimm434 arg4::hisG his1::hisG zfu2::URA3 | BWP17 | This study |
| JFY166 | ura3∆::λimm434 arg4::hisG his1::hisG::pHIS1-ZCF28 zcf28::ARG4ura3∆::λimm434 arg4::hisG his1::hisG zcf28::URA3 | BWP17 | This study |
| JFY176 | ura3∆::λimm434 arg4::hisG his1::hisG::pHIS1 arg81::ARG4ura3∆::λimm434 arg4::hisG his1::hisG arg81::URA3 | BWP17 | This study |
| JFY178 | ura3∆::λimm434 arg4::hisG his1::hisG::pHIS1-ARG81 arg81::ARG4ura3∆::λimm434 arg4::hisG his1::hisG arg81::URA3 | BWP17 | This study |
| DHY2 | ura3∆::λimm434 arg4::hisG his1::hisG::pHIS1 snf5::ARG4ura3∆::λimm434 arg4::hisG his1::hisG snf5::URA3 | BWP17 | This study |
| DHY8 | ura3∆::λimm434 arg4::hisG his1::hisG::pHIS1-SNF5 snf5::ARG4 *ura3∆::λimm434 arg4::hisG his1::hisG snf5::URA3* | BWP17 | This study |
| DHY13 | *ura3∆::λimm434 ARG4:URA3:arg4::hisG his1::hisG::pHIS1 ACE2:pAgTEF1-NAT1-AgTEF1UTR-TDH3-ACE2* ura3∆::λimm434 arg4::hisG his1::hisG: ACE2 | DAY185 | This study |
| DHY14 | *ura3∆::λimm434 ARG4:URA3:arg4::hisG his1::hisG::pHIS1 ACE2:pAgTEF1-NAT1-AgTEF1UTR-TDH3-ACE2* ura3∆::λimm434 arg4::hisG his1::hisG: ACE2 | DAY185 | This study |
| DHY20 | ura3∆::λimm434 arg4::hisG his1::hisG::pHIS1 ACE2:pAgTEF1-NAT1-AgTEF1UTR-TDH3-ACE2 snf5::ARG4ura3∆::λimm434 arg4::hisG his1::hisG ACE2 snf5::URA3 | DHY2 | This study |
| DHY21 | ura3∆::λimm434 arg4::hisG his1::hisG::pHIS1 ACE2:pAgTEF1-NAT1-AgTEF1UTR-TDH3-ACE2 snf5::ARG4 *ura3∆::λimm434 arg4::hisG his1::hisG ACE2 snf5::URA3* | DHY2 | This study |
| DHY26 | ura3∆::λimm434 arg4::hisG his1::hisG::pHIS1 ZAP1:pAgTEF1-NAT1-AgTEF1UTR-TDH3-ZAP1 ada2::Tn7-UAU1 *ura3∆::λimm434 arg4::hisG his1::hisG ZAP1 ada2::Tn7-URA3* | EHY15 | This study |
| DHY27 | ura3∆::λimm434 arg4::hisG his1::hisG::pHIS1 ZAP1:pAgTEF1-NAT1-AgTEF1UTR-TDH3-ZAP1 ada2::Tn7-UAU1 *ura3∆::λimm434 arg4::hisG his1::hisG ZAP1 ada2::Tn7-URA3* | EHY15 | This study |
| DHY28 | ura3∆::λimm434 arg4::hisG his1::hisG::pHIS1 ZAP1:pAgTEF1-NAT1-AgTEF1UTR-TDH3-ZAP1 taf14::Tn7-UAU1 *ura3∆::λimm434 arg4::hisG his1::hisG ZAP1 taf14::Tn7-URA3* | EHY55 | This study |
| DHY29 | ura3∆::λimm434 arg4::hisG his1::hisG::pHIS1 ZAP1:pAgTEF1-NAT1-AgTEF1UTR-TDH3-ZAP1 taf14::Tn7-UAU1 *ura3∆::λimm434 arg4::hisG his1::hisG ZAP1 taf14::Tn7-URA3* | EHY55 | This study |
| DHY30 | ura3∆::λimm434 arg4::hisG his1::hisG::pHIS1 ZAP1:pAgTEF1-NAT1-AgTEF1UTR-TDH3-ZAP1 czf1::Tn7-UAU1 *ura3∆::λimm434 arg4::hisG his1::hisG ZAP1 czf1::Tn7-URA3* | EHY46 | This study |
| DHY31 | ura3∆::λimm434 arg4::hisG his1::hisG::pHIS1 ZAP1:pAgTEF1-NAT1-AgTEF1UTR-TDH3-ZAP1 czf1::Tn7-UAU1 *ura3∆::λimm434 arg4::hisG his1::hisG ZAP1 czf1::Tn7-URA3* | EHY46 | This study |
| DHY47 | ura3∆::λimm434 arg4::hisG his1::hisG::pHIS1 ZAP1:pAgTEF1-NAT1-AgTEF1UTR-TDH3-ZAP1 try5::Tn7-UAU1 *ura3∆::λimm434 arg4::hisG his1::hisG ZAP1 try5::Tn7-URA3* | EHY91 | This study |
| DHY48 | ura3∆::λimm434 arg4::hisG his1::hisG::pHIS1 ZAP1:pAgTEF1-NAT1-AgTEF1UTR-TDH3-ZAP1 try5::Tn7-UAU1 *ura3∆::λimm434 arg4::hisG his1::hisG ZAP1 try5::Tn7-URA3* | EHY91 | This study |
| DHY56 | ura3∆::λimm434 arg4::hisG his1::hisG::pHIS1 ZAP1:pAgTEF1-NAT1-AgTEF1UTR-TDH3-ZAP1 war1::Tn7-UAU1 *ura3∆::λimm434 arg4::hisG his1::hisG ZAP1 war1::Tn7-URA3* | EHY73 | This study |
| DHY57 | ura3∆::λimm434 arg4::hisG his1::hisG::pHIS1 ZAP1:pAgTEF1-NAT1-AgTEF1UTR-TDH3-ZAP1 war1::Tn7-UAU1 *ura3∆::λimm434 arg4::hisG his1::hisG ZAP1 war1::Tn7-URA3* | EHY73 | This study |
| JFY251 | ura3∆::λimm434 arg4::hisG his1::hisG::pHIS1 ZAP1:pAgTEF1-NAT1-AgTEF1UTR-TDH3-ZAP1 try3::Tn7-UAU1 *ura3∆::λimm434 arg4::hisG his1::hisG ZAP1 try3::Tn7-URA3* | EHY30 | This study |
| JFY252 | ura3∆::λimm434 arg4::hisG his1::hisG::pHIS1 ZAP1:pAgTEF1-NAT1-AgTEF1UTR-TDH3-ZAP1 try3::Tn7-UAU1 *ura3∆::λimm434 arg4::hisG his1::hisG ZAP1 try3::Tn7-URA3* | EHY30 | This study |
| JFY253 | *ura3∆::λimm434 arg4::hisG his1::hisG::pHIS1 ZAP1:pAgTEF1-NAT1-AgTEF1UTR-TDH3-ZAP1 fgr27::Tn7-UAU1 ura3∆::λimm434 arg4::hisG his1::hisG ZAP1 fgr27::Tn7-URA3* | EHY40 | This study |
| JFY254 | *ura3∆::λimm434 arg4::hisG his1::hisG::pHIS1 ZAP1:pAgTEF1-NAT1-AgTEF1UTR-TDH3-ZAP1 fgr27::Tn7-UAU1 ura3∆::λimm434 arg4::hisG his1::hisG ZAP1 fgr27::Tn7-URA3* | EHY40 | This study |
| JFY255 | ura3∆::λimm434 arg4::hisG his1::hisG::pHIS1 ZAP1:pAgTEF1-NAT1-AgTEF1UTR-TDH3-ZAP1 arg81::ARG4 *ura3∆::λimm434 arg4::hisG his1::hisG ZAP1 arg81::URA3* | JFY176 | This study |
| JFY256 | ura3∆::λimm434 arg4::hisG his1::hisG::pHIS1 ZAP1:pAgTEF1-NAT1-AgTEF1UTR-TDH3-ZAP1 arg81::ARG4 *ura3∆::λimm434 arg4::hisG his1::hisG ZAP1 arg81::URA3* | JFY176 | This study |
| JFY257 | ura3∆::λimm434 arg4::hisG his1::hisG::pHIS1 ZAP1:pAgTEF1-NAT1-AgTEF1UTR-TDH3-ZAP1 zcf8::Tn7-UAU1 *ura3∆::λimm434 arg4::hisG his1::hisG ZAP1 zcf8::Tn7-URA3* | EHY76 | This study |
| JFY258 | ura3∆::λimm434 arg4::hisG his1::hisG::pHIS1 ZAP1:pAgTEF1-NAT1-AgTEF1UTR-TDH3-ZAP1 zcf8::Tn7-UAU1 *ura3∆::λimm434 arg4::hisG his1::hisG ZAP1 zcf8::Tn7-URA3* | EHY76 | This study |
| JFY259 | ura3∆::λimm434 arg4::hisG his1::hisG::pHIS1 ZAP1:pAgTEF1-NAT1-AgTEF1UTR-TDH3-ZAP1 znc1::Tn7-UAU1 *ura3∆::λimm434 arg4::hisG his1::hisG ZAP1 znc1::Tn7-URA3* | EHY34 | This study |
| JFY260 | ura3∆::λimm434 arg4::hisG his1::hisG::pHIS1 ZAP1:pAgTEF1-NAT1-AgTEF1UTR-TDH3-ZAP1 znc1::Tn7-UAU1ura3∆::λimm434 arg4::hisG his1::hisG ZAP1 znc1::Tn7-URA3 | EHY34 | This study |
| JFY261 | ura3∆::λimm434 arg4::hisG his1::hisG::pHIS1 ZAP1:pAgTEF1-NAT1-AgTEF1UTR-TDH3-ZAP1 zcf28::Tn7-UAU1 *ura3∆::λimm434 arg4::hisG his1::hisG ZAP1 zcf28::Tn7-URA3* | JFY144 | This study |
| JFY262 | ura3∆::λimm434 arg4::hisG his1::hisG::pHIS1 ZAP1:pAgTEF1-NAT1-AgTEF1UTR-TDH3-ZAP1 zcf28::Tn7-UAU1 *ura3∆::λimm434 arg4::hisG his1::hisG ZAP1 zcf28::Tn7-URA3* | JFY144 | This study |
| JFY267 | ura3∆::λimm434 arg4::hisG his1::hisG::pHIS1 ZAP1:pAgTEF1-NAT1-AgTEF1UTR-TDH3-ZAP1 bcr1::ARG4 *ura3∆::λimm434 arg4::hisG his1::hisG ZAP1 bcr1::URA3* | CJN702 | This study |
| JFY268 | ura3∆::λimm434 arg4::hisG his1::hisG::pHIS1 ZAP1:pAgTEF1-NAT1-AgTEF1UTR-TDH3-ZAP1 bcr1::ARG4 *ura3∆::λimm434 arg4::hisG his1::hisG ZAP1 bcr1::URA3* | CJN702 | This study |
| JFY270 | ura3∆::λimm434 arg4::hisG his1::hisG::pHIS1 ZAP1:pAgTEF1-NAT1-AgTEF1UTR-TDH3-ZAP1 met4::Tn7-UAU1 *ura3∆::λimm434 arg4::hisG his1::hisG ZAP1 met4::Tn7-URA3* | EHY106 | This study |
| JFY271 | ura3∆::λimm434 arg4::hisG his1::hisG::pHIS1 ZAP1:pAgTEF1-NAT1-AgTEF1UTR-TDH3-ZAP1 met4::Tn7-UAU1 *ura3∆::λimm434 arg4::hisG his1::hisG ZAP1 met4::Tn7-URA3* | EHY106 | This study |
| JFY272 | ura3∆::λimm434 arg4::hisG his1::hisG::pHIS1 ZAP1:pAgTEF1-NAT1-AgTEF1UTR-TDH3-ZAP1 try4::Tn7-UAU1 *ura3∆::λimm434 arg4::hisG his1::hisG ZAP1 try4::Tn7-URA3* | EHY27 | This study |
| JFY273 | ura3∆::λimm434 arg4::hisG his1::hisG::pHIS1 ZAP1:pAgTEF1-NAT1-AgTEF1UTR-TDH3-ZAP1 try4::Tn7-UAU1 *ura3∆::λimm434 arg4::hisG his1::hisG ZAP1 try4::Tn7-URA3* | EHY27 | This study |
| JFY276 | ura3∆::λimm434 arg4::hisG his1::hisG::pHIS1 ZAP1:pAgTEF1-NAT1-AgTEF1UTR-TDH3-ZAP1 suc1::Tn7-UAU1 *ura3∆::λimm434 arg4::hisG his1::hisG ZAP1 suc1::Tn7-URA3* | EHY85 | This study |
| JFY277 | ura3∆::λimm434 arg4::hisG his1::hisG::pHIS1 ZAP1:pAgTEF1-NAT1-AgTEF1UTR-TDH3-ZAP1 suc1::Tn7-UAU1 *ura3∆::λimm434 arg4::hisG his1::hisG ZAP1 suc1::Tn7-URA3* | EHY85 | This study |
| JFY280 | ura3∆::λimm434 arg4::hisG his1::hisG::pHIS1 ZAP1:pAgTEF1-NAT1-AgTEF1UTR-TDH3-ZAP1 zcf34::Tn7-UAU1 *ura3∆::λimm434 arg4::hisG his1::hisG ZAP1 zcf34::Tn7-URA3* | EHY108 | This study |
| JFY281 | ura3∆::λimm434 arg4::hisG his1::hisG::pHIS1 ZAP1:pAgTEF1-NAT1-AgTEF1UTR-TDH3-ZAP1 zcf34::Tn7-UAU1 *ura3∆::λimm434 arg4::hisG his1::hisG ZAP1 zcf34::Tn7-URA3* | EHY108 | This study |
| JFY285 | *ura3∆::λimm434 arg4::hisG his1::hisG::pHIS1 ZAP1:pAgTEF1-NAT1-AgTEF1UTR-TDH3-ZAP1 zcf31::Tn7-UAU1 ura3∆::λimm434 arg4::hisG his1::hisG ZAP1 zcf31::Tn7-URA3* | EHY70 | This study |
| JFY286 | *ura3∆::λimm434 arg4::hisG his1::hisG::pHIS1 ZAP1:pAgTEF1-NAT1-AgTEF1UTR-TDH3-ZAP1 zcf31::Tn7-UAU1 ura3∆::λimm434 arg4::hisG his1::hisG ZAP1 zcf31::Tn7-URA3* | EHY70 | This study |
| JFY300 | ura3∆::λimm434 arg4::hisG his1::hisG::pHIS1 ZAP1:pAgTEF1-NAT1-AgTEF1UTR-TDH3-ZAP1 dal81::Tn7-UAU1 *ura3∆::λimm434 arg4::hisG his1::hisG ZAP1 dal81::Tn7-URA3* | EHY67 | This study |
| JFY301 | ura3∆::λimm434 arg4::hisG his1::hisG::pHIS1 ZAP1:pAgTEF1-NAT1-AgTEF1UTR-TDH3-ZAP1 dal81::Tn7-UAU1 *ura3∆::λimm434 arg4::hisG his1::hisG ZAP1 dal81::Tn7-URA3* | EHY67 | This study |
| JFY307 | ura3∆::λimm434 arg4::hisG his1::hisG::pHIS1 ZAP1:pAgTEF1-NAT1-AgTEF1UTR-TDH3-ZAP1 leu3::Tn7-UAU1 *ura3∆::λimm434 arg4::hisG his1::hisG ZAP1 leu3::Tn7-URA3* | EHY88 | This study |
| JFY308 | ura3∆::λimm434 arg4::hisG his1::hisG::pHIS1 ZAP1:pAgTEF1-NAT1-AgTEF1UTR-TDH3-ZAP1 leu3::Tn7-UAU1 *ura3∆::λimm434 arg4::hisG his1::hisG ZAP1 leu3::Tn7-URA3* | EHY88 | This study |
| JFY309 | ura3∆::λimm434 arg4::hisG his1::hisG::pHIS1 ZAP1:pAgTEF1-NAT1-AgTEF1UTR-TDH3-ZAP1 zfu2::ARG1 *ura3∆::λimm434 arg4::hisG his1::hisG ZAP1 zfu2::URA3* | BWP17 | This study |
| JFY310 | ura3∆::λimm434 arg4::hisG his1::hisG::pHIS1 ZAP1:pAgTEF1-NAT1-AgTEF1UTR-TDH3-ZAP1 zfu2::ARG1ura3∆::λimm434 arg4::hisG his1::hisG ZAP1 zfu2::URA3 | BWP17 | This study |
| JFY312 | ura3∆::λimm434 arg4::hisG his1::hisG::pHIS1 ZAP1:pAgTEF1-NAT1-AgTEF1UTR-TDH3-ZAP1 try6::Tn7-UAU1 *ura3∆::λimm434 arg4::hisG his1::hisG ZAP1 try6::Tn7-URA3* | EHY103 | This study |
| JFY313 | ura3∆::λimm434 arg4::hisG his1::hisG::pHIS1 ZAP1:pAgTEF1-NAT1-AgTEF1UTR-TDH3-ZAP1 try6::Tn7-UAU1 *ura3∆::λimm434 arg4::hisG his1::hisG ZAP1 try6::Tn7-URA3* | EHY103 | This study |
| JFY319 | ura3∆::λimm434 arg4::hisG his1::hisG::pHIS1 ZAP1:pAgTEF1-NAT1-AgTEF1UTR-TDH3-ZAP1 not3::Tn7-UAU1 *ura3∆::λimm434 arg4::hisG his1::hisG ZAP1 not3::Tn7-URA3* | EHY79 | This study |
| JFY320 | ura3∆::λimm434 arg4::hisG his1::hisG::pHIS1 ZAP1:pAgTEF1-NAT1-AgTEF1UTR-TDH3-ZAP1 not3::Tn7-UAU1 *ura3∆::λimm434 arg4::hisG his1::hisG ZAP1 not3::Tn7-URA3* | EHY79 | This study |
| JFY322 | ura3∆::λimm434 arg4::hisG his1::hisG::pHIS1 ZAP1:pAgTEF1-NAT1-AgTEF1UTR-TDH3-ZAP1 snf5::ARG1 *ura3∆::λimm434 arg4::hisG his1::hisG ZAP1 snf5::URA3* | DHY2 | This study |
| JFY323 | ura3∆::λimm434 arg4::hisG his1::hisG::pHIS1 ZAP1:pAgTEF1-NAT1-AgTEF1UTR-TDH3-ZAP1 snf5::ARG1ura3∆::λimm434 arg4::hisG his1::hisG ZAP1 snf5::URA3 | DHY2 | This study |
| JFY328 | ura3∆::λimm434 arg4::hisG his1::hisG::pHIS1 ZAP1:pAgTEF1-NAT1-AgTEF1UTR-TDH3-ZAP1 fcr3::Tn7-UAU1 *ura3∆::λimm434 arg4::hisG his1::hisG ZAP1 fcr3::Tn7-URA3* | EHY61 | This study |
| JFY329 | ura3∆::λimm434 arg4::hisG his1::hisG::pHIS1 ZAP1:pAgTEF1-NAT1-AgTEF1UTR-TDH3-ZAP1 fcr3::Tn7-UAU1 *ura3∆::λimm434 arg4::hisG his1::hisG ZAP1 fcr3::Tn7-URA3* | EHY61 | This study |
| JFY333 | ura3∆::λimm434 arg4::hisG his1::hisG::pHIS1 ZAP1:pAgTEF1-NAT1-AgTEF1UTR-TDH3-ZAP1 zcf39::Tn7-UAU1 *ura3∆::λimm434 arg4::hisG his1::hisG ZAP1 zcf39::Tn7-URA3* | EHY43 | This study |
| JFY334 | ura3∆::λimm434 arg4::hisG his1::hisG::pHIS1 ZAP1:pAgTEF1-NAT1-AgTEF1UTR-TDH3-ZAP1 zcf39::Tn7-UAU1 *ura3∆::λimm434 arg4::hisG his1::hisG ZAP1 zcf39::Tn7-URA3* | EHY43 | This study |
| JFY335 | ura3∆::λimm434 arg4::hisG his1::hisG::pHIS1 ZAP1:pAgTEF1-NAT1-AgTEF1UTR-TDH3-ZAP1 crz2::ARG1 *ura3∆::λimm434 arg4::hisG his1::hisG ZAP1 crz2::URA3* | DAY951 | This study |
| JFY336 | ura3∆::λimm434 arg4::hisG his1::hisG::pHIS1 ZAP1:pAgTEF1-NAT1-AgTEF1UTR-TDH3-ZAP1 crz2::ARG1 *ura3∆::λimm434 arg4::hisG his1::hisG ZAP1 crz2::URA3* | DAY951 | This study |
| JFY337 | ura3∆::λimm434 arg4::hisG his1::hisG::pHIS1 ZAP1:pAgTEF1-NAT1-AgTEF1UTR-TDH3-ZAP1 try2::Tn7-UAU1 *ura3∆::λimm434 arg4::hisG his1::hisG ZAP1 try2::Tn7-URA3* | EHY97 | This study |
| JFY338 | ura3∆::λimm434 arg4::hisG his1::hisG::pHIS1 ZAP1:pAgTEF1-NAT1-AgTEF1UTR-TDH3-ZAP1 try2::Tn7-UAU1 *ura3∆::λimm434 arg4::hisG his1::hisG ZAP1 try2::Tn7-URA3* | EHY97 | This study |
| JFY339 | ura3∆::λimm434 arg4::hisG his1::hisG::pHIS1 ZAP1:pAgTEF1-NAT1-AgTEF1UTR-TDH3-ZAP1 uga33::Tn7-UAU1 *ura3∆::λimm434 arg4::hisG his1::hisG ZAP1 uga33::Tn7-URA3* | EHY8 | This study |
| JFY340 | ura3∆::λimm434 arg4::hisG his1::hisG::pHIS1 ZAP1:pAgTEF1-NAT1-AgTEF1UTR-TDH3-ZAP1 uga33::Tn7-UAU1 *ura3∆::λimm434 arg4::hisG his1::hisG ZAP1 uga33::Tn7-URA3* | EHY8 | This study |
| JFY348 | ura3∆::λimm434 ARG4:URA3:arg4::hisG his1::hisG::pHIS1 ZAP1:pAgTEF1-NAT1-AgTEF1UTR-TDH3-ZAP1 *ura3∆::λimm434 arg4::hisG his1::hisG ZAP1* | DAY185 | This study |
| JFY349 | ura3∆::λimm434 ARG4:URA3:arg4::hisG his1::hisG::pHIS1 ZAP1:pAgTEF1-NAT1-AgTEF1UTR-TDH3-ZAP1 *ura3∆::λimm434 arg4::hisG his1::hisG ZAP1* | DAY185 | This study |
| JFY350 | ura3∆::λimm434 arg4::hisG his1::hisG::pHIS1 ace2::ARG4 ZAP1:pAgTEF1-NAT1-AgTEF1UTR-TDH3-ZAP1 *ura3∆::λimm434 arg4::hisG his1::hisG ace2::URA3 ZAP1* | CTN85 | This study |
| JFY351 | ura3∆::λimm434 arg4::hisG his1::hisG::pHIS1 ace2::ARG4 ZAP1:pAgTEF1-NAT1-AgTEF1UTR-TDH3-ZAP1 *ura3∆::λimm434 arg4::hisG his1::hisG ace2::URA3 ZAP1* | CTN85 | This study |
| AMP271 | *MATa ura3 leu2-hisG trp1-hisG lys2 ho-LYS2 his1-229S gal80-LEU2 ime1D12-TRP1* |  |  |
| CW431 | ura3∆::λimm434 arg4::hisG his1::hisG::pHIS1-SUC1 suc1::Tn7-UAU1 *ura3∆::λimm434 arg4::hisG his1::hisG suc1::Tn7-URA3* | RLS3 | This study |
| CW432 | ura3∆::λimm434 arg4::hisG his1::hisG::pHIS1-SUC1 suc1::Tn7-UAU1 *ura3∆::λimm434 arg4::hisG his1::hisG suc1::Tn7-URA3* | RLS3 | This study |
| CW434 | ura3∆::λimm434 arg4::hisG his1::hisG::pHIS1-TRY2 try2::Tn7-UAU1 *ura3∆::λimm434 arg4::hisG his1::hisG try2::Tn7-URA3* | SFY7 | This study |
| CW437 | ura3∆::λimm434 arg4::hisG his1::hisG::pHIS1-TRY3 try3::Tn7-UAU1 *ura3∆::λimm434 arg4::hisG his1::hisG try3::Tn7-URA3* | SFY42 | This study |
| CW438 | ura3∆::λimm434 arg4::hisG his1::hisG::pHIS1-TRY3 try3::Tn7-UAU1 *ura3∆::λimm434 arg4::hisG his1::hisG try3::Tn7-URA3* | SFY42 | This study |
| CW440 | ura3∆::λimm434 arg4::hisG his1::hisG::pHIS1-TRY4 try4::Tn7-UAU1 *ura3∆::λimm434 arg4::hisG his1::hisG try4::Tn7-URA3* | CJN809 | This study |
| CW441 | ura3∆::λimm434 arg4::hisG his1::hisG::pHIS1-TRY4 try4::Tn7-UAU1 *ura3∆::λimm434 arg4::hisG his1::hisG try4::Tn7-URA3* | CJN809 | This study |
| CW443 | ura3∆::λimm434 arg4::hisG his1::hisG::pHIS1-TRY5 try5::Tn7-UAU1 *ura3∆::λimm434 arg4::hisG his1::hisG try5::Tn7-URA3* | RLS50 | This study |
| CW445 | ura3∆::λimm434 arg4::hisG his1::hisG::pHIS1-TRY6 try6::Tn7-UAU1 *ura3∆::λimm434 arg4::hisG his1::hisG try6::Tn7-URA3* | SFY10 | This study |
| CW446 | ura3∆::λimm434 arg4::hisG his1::hisG::pHIS1-TRY6 try6::Tn7-UAU1 *ura3∆::λimm434 arg4::hisG his1::hisG try6::Tn7-URA3* | SFY10 | This study |
| CW453 | ura3∆::λimm434 arg4::hisG his1::hisG::pHIS1-DAL81 dal81::Tn7-UAU1 *ura3∆::λimm434 arg4::hisG his1::hisG dal81::Tn7-URA3* | DSY3414-1 | This study |
| CW454 | ura3∆::λimm434 arg4::hisG his1::hisG::pHIS1-DAL81 dal81::Tn7-UAU1 *ura3∆::λimm434 arg4::hisG his1::hisG dal81::Tn7-URA3* | DSY3414-1 | This study |
| CW456 | ura3∆::λimm434 arg4::hisG his1::hisG::pHIS1-ZCF34 zcf34::Tn7-UAU1 *ura3∆::λimm434 arg4::hisG his1::hisG zcf34::Tn7-URA3* | CJN548 | This study |
| CW457 | ura3∆::λimm434 arg4::hisG his1::hisG::pHIS1-ZCF34 zcf34::Tn7-UAU1ura3∆::λimm434 arg4::hisG his1::hisG zcf34::Tn7-URA3 | CJN548 | This study |
| CW459 | ura3∆::λimm434 arg4::hisG his1::hisG::pHIS1-ZCF8 zcf8::Tn7-UAU1 *ura3∆::λimm434 arg4::hisG his1::hisG zcf8::Tn7-URA3* | DSY3447-11 | This study |
| CW460 | ura3∆::λimm434 arg4::hisG his1::hisG::pHIS1-ZCF8 zcf8::Tn7-UAU1 *ura3∆::λimm434 arg4::hisG his1::hisG zcf8::Tn7-URA3* | DSY3447-11 | This study |
| CW462 | ura3∆::λimm434 arg4::hisG his1::hisG::pHIS1-FCR3 fcr3::Tn7-UAU1 *ura3∆::λimm434 arg4::hisG his1::hisG fcr3::Tn7-URA3* | CJN926 | This study |
| CW463 | ura3∆::λimm434 arg4::hisG his1::hisG::pHIS1-FCR3 fcr3::Tn7-UAU1 *ura3∆::λimm434 arg4::hisG his1::hisG fcr3::Tn7-URA3* | CJN926 | This study |
| CW465 | ura3∆::λimm434 arg4::hisG his1::hisG::pHIS1-UGA33 uga33::Tn7-UAU1 *ura3∆::λimm434 arg4::hisG his1::hisG uga33::Tn7-URA3* | CJN571 | This study |
| CW466 | ura3∆::λimm434 arg4::hisG his1::hisG::pHIS1-UGA33 uga33::Tn7-UAU1 *ura3∆::λimm434 arg4::hisG his1::hisG uga33::Tn7-URA3* | CJN571 | This study |
| VIC1145 | ura3∆::λimm434 arg4::hisG his1::hisG::pHIS1 ada2::ARG4 *ura3∆::λimm434 arg4::hisG his1::hisG ada2::URA3* | BWP17 | {Bruno, 2006 #4} |
| VIC1151 | ura3∆::λimm434 arg4::hisG his1::hisG::pHIS1 ada2::ARG4 *ura3∆::λimm434 arg4::hisG his1::hisG ada2::URA3* | BWP17 | {Bruno, 2006 #4} |
| VIC1197 | ura3∆::λimm434 arg4::hisG his1::hisG::pHIS1-ADA2 ada2::ARG4 *ura3∆::λimm434 arg4::hisG his1::hisG ada2::URA3* | BWP17 | {Bruno, 2006 #4} |
